# Gut Microbiome of the Canadian Arctic Inuit

**DOI:** 10.1128/mSphere.00297-16

**Published:** 2017-01-04

**Authors:** Catherine Girard, Nicolas Tromas, Marc Amyot, B. Jesse Shapiro

**Affiliations:** a Centre for Northern Studies, Département de sciences biologiques, Université de Montréal, Montreal, Quebec, Canada; b Département de sciences biologiques, Université de Montréal, Montreal, Quebec, Canada; Arizona State University

**Keywords:** *Akkermansia*, alpha diversity, Inuit microbiome, oligotyping, *Prevotella*, traditional diet, Western diet

## Abstract

Diet is a major determinant of community composition in the human gut microbiome, and “traditional” diets have been associated with distinct and highly diverse communities, compared to Western diets. However, most traditional diets studied have been those of agrarians and hunter-gatherers consuming fiber-rich diets. In contrast, the Inuit of the Canadian Arctic have been consuming a traditional diet low in carbohydrates and rich in animal fats and protein for thousands of years. We hypothesized that the Inuit diet and lifestyle would be associated with a distinct microbiome. We used deep sequencing of the 16S rRNA gene to compare the gut microbiomes of Montrealers with a Western diet to those of the Inuit consuming a range of traditional and Western diets. At the overall microbial community level, the gut microbiomes of Montrealers and Inuit were indistinguishable and contained similar levels of microbial diversity. However, we observed significant differences in the relative abundances of certain microbial taxa down to the subgenus level using oligotyping. For example, *Prevotella* spp., which have been previously associated with high-fiber diets, were enriched in Montrealers and among the Inuit consuming a Western diet. The gut microbiomes of Inuit consuming a traditional diet also had significantly less genetic diversity within the *Prevotella* genus, suggesting that a low-fiber diet might not only select against *Prevotella* but also reduce its diversity. Other microbes, such as *Akkermansia*, were associated with geography as well as diet, suggesting limited dispersal to the Arctic. Our report provides a snapshot of the Inuit microbiome as Western-like in overall community structure but distinct in the relative abundances and diversity of certain genera and strains.

**IMPORTANCE** Non-Western populations have been shown to have distinct gut microbial communities shaped by traditional diets. The hitherto-uncharacterized microbiome of the Inuit may help us to better understand health risks specific to this population such as diabetes and obesity, which increase in prevalence as many Inuit transition to a Western diet. Here we show that even Inuit consuming a mostly traditional diet have a broadly Western-like microbiome. This suggests that similarities between the Inuit diet and the Western diet (low fiber, high fat) may lead to a convergence of community structures and diversity. However, certain species and strains of microbes have significantly different levels of abundance and diversity in the Inuit, possibly driven by differences in diet. Furthermore, the Inuit diet provides an exception to the correlation between traditional diets and high microbial diversity, potentially due to their transitioning diet. Knowledge of the Inuit microbiome may provide future resources for interventions and conservation of Inuit heritage.

## Abstract (precis)

Non-Western populations have been shown to have distinct gut microbial communities shaped by traditional diets. The hitherto-uncharacterized microbiome of the Inuit may help us to better understand health risks specific to this population such as diabetes and obesity, which increase in prevalence as many Inuit transition to a Western diet. Here we show that even Inuit consuming a mostly traditional diet have a broadly Western-like microbiome. This suggests that similarities between the Inuit diet and the Western diet (low fiber, high fat) may lead to a convergence of community structures and diversity. However, certain species and strains of microbes have significantly different levels of abundance and diversity in the Inuit, possibly driven by differences in diet. Furthermore, the Inuit diet provides an exception to the correlation between traditional diets and high microbial diversity, potentially due to their transitioning diet. Knowledge of the Inuit microbiome may provide future resources for interventions and conservation of Inuit heritage.

## INTRODUCTION

The human gut microbiome is a complex ecosystem of microbes that contribute to host immunity, nutrition, and behavior (1–3) and varies with diet, lifestyle, and disease (4–7). The gut microbiome is an important source of genetic and metabolic variation across human populations (8, 9). Diet is one of the main drivers of community structure of the intestinal microbial community (4), and much effort has been put into characterizing the microbiome of populations with contrasting dietary habits. Studies comparing microbiomes of Westerners to those of agrarian or hunter-gatherer populations found significant differences associated with their contrasting diets (8, 10–13). For example, *Prevotella* and *Xylanibacter* were associated with a diet rich in indigestible polysaccharides due to their fermentative abilities (11). Short-term consumption of an extremely animal-rich diet (composed entirely of animal products) was experimentally shown to significantly alter the human gut microbiome, increasing the relative abundances of *Bacteroides*, *Bilophila*, and *Alistipes* while reducing the abundance of polysaccharide-degrading *Firmicutes* (4). However, animal-rich diets have yet to be explored in more realistic, natural contexts, such as the Inuit inhabiting the Arctic regions of the world (14).

The traditionally nomadic culture of the Inuit is based on the hunt and gathering of food from the environment (15). The Arctic environment has shaped the traditional Inuit diet, which includes many land and marine mammals, such as caribou, musk-ox, seal, whale, and fish, and this traditional diet has been consumed for hundreds if not thousands of years (16). Meat is often consumed raw and, occasionally, frozen, dried, or cooked. Like many other indigenous peoples around the world, the Inuit are undergoing a rapid transition away from their traditional diet toward a more Western diet (15, 17). For the Inuit, major lifestyle changes in the last hundred years (including settlement into permanent communities) have favored a shift toward processed store-bought foods shipped from the south and away from traditional food, leading to lower micronutrient intakes (15, 17, 18). This shift could impact the gut microbiome of the Inuit, with potential health consequences. The modern Inuit diet is therefore different from the ancestral diet, with Western food becoming more and more popular. The Inuit may thus be an example of a population nearing the end of its transition toward the Western diet (15, 17).

The Inuit have a unique set of health risks, many of which could be modulated by the microbiome. For instance, obesity rates in northern Canada currently exceed the national average (17). However, the consequences of obesity may be different for the Inuit: a study comparing the Inuit to Europeans and southern Canadians found that at every body mass index (BMI) level, the Inuit had lower blood pressure and lipid levels than their Western counterparts (19). It is currently unclear how the Inuit microbiome might contribute to these different clinical manifestations of obesity and other Inuit-specific health risks.

We hypothesized that modern Inuit harbor a distinct gut microbiome, associated with traditional diet. We compared the gut microbiome of an Inuit population with a range of traditional and Western diets to those of individuals from Montreal, Canada, adhering to a typical Western diet. We found that at the broad scale of the entire gut microbiome community, the Inuit resemble Montrealers in both community composition and diversity. However, we identified subtle but significant differences in the relative abundances of several microbial taxa, driven by a combination of dietary and environmental factors.

## RESULTS

### Study populations.

To compare characteristics of gut microbial diversity and community composition, we subjected 16S amplicons from stool samples to deep sequencing. We sequenced samples from 19 adults (16 Inuit, 2 individuals of European descent, and 1 person of mixed heritage) from an Arctic community in the Canadian territory of Nunavut and from 26 adults of European descent from Montreal—a metropolitan area at a temperate latitude (Materials and Methods; see also Tables S1A and B in the supplemental material). The majority of participants from Nunavut adhered to a modern traditional Inuit diet and frequently consumed raw game, especially sea mammals (see Fig. S1 in the supplemental material and Table S1A). The Inuit diet is limited in plant-derived foods and is enriched in animal protein (17, 20) and is an excellent source of vitamins, minerals, and micronutrients (17). For the purposes of this study, we defined a traditional diet as one in which traditional meats were consumed daily or multiple times a week (Table S1C). This definition does not exclude individuals who also consumed imported or packaged foods. However, three Nunavut participants who never or only occasionally consumed the traditional Inuit diet (once per week or less often), along with participants from Montreal, were classified as having a fully Western diet. The average body mass index (BMI) of participants in Nunavut (28.1 ± 7.0 kg·m^−2^) was significantly higher than in Montreal (23.4 ± 3.5 kg·m^−2^) (*t* test, *P* <5 × 10^−3^; Table S1B).

10.1128/mSphere.00297-16.1 TABLE S1  (A) Traditional food consumption in the Nunavut cohort. Examples of frequently consumed traditional foods are shown on the left. On the right, respondent data are binned according to the frequency of their traditional food consumption, ranging from never to infrequently (once a week or less) to frequently (at least twice a week) to every day. (B) Characteristics of study participants. The Inuit and Western cohorts had similar gender and age representations (*t* test, *P > 0.05*). Inuit participants had higher BMI values (*t* test, *P* < 0.005) and more varied ethnic representation. We collected samples from 26 Nunavut participants, 19 of which were successfully sequenced. A total of 33 samples were collected in Montreal, 26 of which passed our quality filters. Sampling was restricted to individuals over 20 years of age who had not used antibiotics in the 3 months preceding sampling. Eight Nunavut participants shared households (4 households). (C) Dietary information collected during surveys, showing how frequency was converted into bins (Inuit and Western diets). The table also includes BMI, ethnicity, and gender information. (D) OTUs enriched in Nunavut or Montreal samples and identified with DESeq2. *P* values were corrected for multiple comparisons using the false-discovery rate (FDR). baseMean data show average normalized count values divided by size factor; lfcSE data show standard error estimates for log2FC. A negative Log2FC result indicates enrichment in the Montreal diet. (E) OTUs enriched in the Inuit or Western diet samples across all participants, identified with DESeq2. *P* values were corrected for multiple comparisons using the false-discovery rate (FDR). baseMean data show average normalized count values divided by size factor; lfcSE data show standard error estimates for log2FC. A negative Log2FC result indicates enrichment in the Inuit diet. (F) OTUs enriched in samples from individuals with a BMI of <25 and >25 across all participants, identified with DESeq2. *P* values were corrected for multiple comparisons using the false-discovery rate (FDR). baseMean data show average normalized count values divided by size factor; lfcSE data show standard error estimates for log2FC. A negative Log2FC result indicates enrichment in samples from individuals with a BMI of <25. (G) LEfSe analysis identified biomarkers for Nunavut (blue) and Montreal (yellow) and Western (green) and Inuit (purple) diets across the whole data set and biomarkers of the Western diet identified in Nunavut participants only (red). Biomarkers of high BMI (dark purple) and low BMI (dark blue) are also reported. Taxa are ranked according to their estimated effect size (LDA score). Asterisks denote biomarkers of Nunavut also identified as biomarkers of the Inuit diet; crosses denote biomarkers of Nunavut, of the Inuit diet, and of BMI >25. (H) LefSe analysis of *Akkermensia* and *Prevotella* oligotypes. Download TABLE S1, XLSX file, 0.1 MB. Copyright © 2017 Girard et al. 2017 Girard et al. This content is distributed under the terms of the Creative Commons Attribution 4.0 International license.

10.1128/mSphere.00297-16.2 FIG S1  (A and B) The Inuit community sampled is located in the Canadian Arctic (74°41′51″N, 94°49′56″W) in the region indicated in the map (A) and photograph (B) and has a population of 214 (2011 Canadian Census). (C and D) The traditional Inuit diet includes various land and marine animals such as caribou (C) and seals (D). Download FIG S1, PDF file, 1 MB. Copyright © 2017 Girard et al. 2017 Girard et al. This content is distributed under the terms of the Creative Commons Attribution 4.0 International license.

### Similar levels of alpha diversity in the Inuit and Western microbiomes.

**FIG 1:**
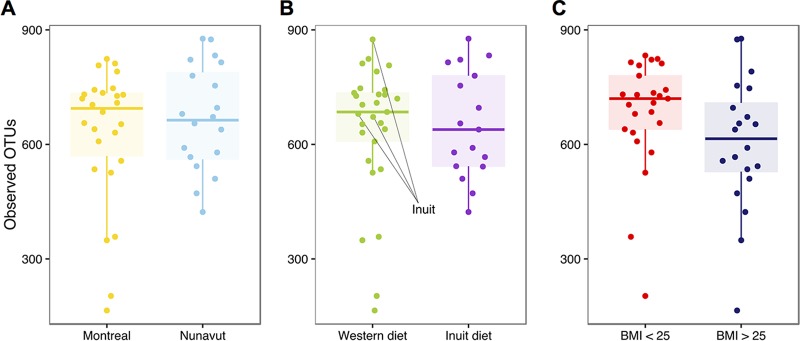
Similar levels of microbiome diversity across diet and geography. We observed no significant differences in levels of microbial taxonomic diversity in samples compared by geography (A), diet (B), or BMI (C) (Mann-Whitney test; *P* > 0.05). See Fig. S2 for other diversity indices. OTUs were identified by open reference OTU picking (see Materials and Methods). Box plots show the medians, and whiskers show 25% and 75% quartiles.

In previous studies of the gut microbiome, greater diversity has consistently been observed in agrarians and hunter-gatherers than in members of Western industrialized populations (8, 10–13). We therefore asked whether the Nunavut population or the traditional Inuit diet was associated with high or low microbiome diversity. Using a variety of diversity indices, we found no significant differences in the observed numbers of bacterial operational taxonomic units (OTUs, defined at 97% nucleotide identity; Materials and Methods) or other alpha diversity metrics between Montreal and Nunavut (Mann-Whitney test; *P* > 0.05) (Fig. 1A; see also Fig. S2). No differences in diversity were observed by diet (Fig. 1B; Fig. S2). Restricting the dietary comparison to Nunavut residents only, and thereby controlling for geography, there was a tendency toward lower diversity in participants consuming an Inuit diet, although the results were not statistically significant (Fig. S2C). Ethnicity did not have a measurable impact on alpha diversity (Fig. S2E).

10.1128/mSphere.00297-16.3 FIG S2  (A to D) Comparison of alpha diversity metrics between populations (A), ethnic groups (B), and diets among all participants (C) and diet only among Nunavut participants (D) (Mann-Whitney test; *P* > 0.05). (E) Only BMI levels had significant differences, with lower BMIs (i.e., leaner individuals) being more diverse in Shannon and Simpson diversity metrics (Mann-Whitney test; *P* < 0.05). See Text S1 for descriptions of metrics. Download FIG S2, PDF file, 0.3 MB. Copyright © 2017 Girard et al. 2017 Girard et al. This content is distributed under the terms of the Creative Commons Attribution 4.0 International license.

10.1128/mSphere.00297-16.9 TEXT S1  Supplementary Materials and Methods. Download TEXT S1, DOCX file, 0.1 MB. Copyright © 2017 Girard et al. 2017 Girard et al. This content is distributed under the terms of the Creative Commons Attribution 4.0 International license.

**FIG 2:**
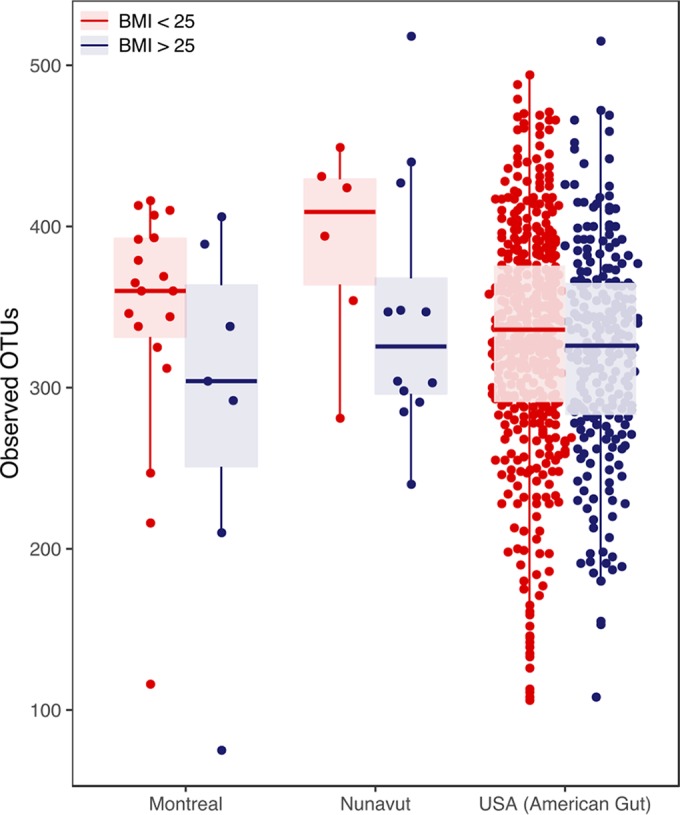
Comparison of levels of microbiome diversity by BMI, stratified by geography. To put our data in the context of a larger study, we performed closed-reference OTU picking to compare OTU counts across our data set and 1,000 random samples from the American Gut project. In all geographic regions (Montreal, Nunavut, and the United States), lean individuals (BMI = <25) had slightly higher diversity (number of observed OTUs) than overweight individuals (BMI >25), but the differences were not significant for any of the comparisons (Mann-Whitney test; *P* > 0.05). Box plots show the median, and whiskers show 25% and 75% quartiles.

On average, overweight individuals (BMI of >25) had lower Shannon and Simpson diversity values (Manny-Whitney test; *P* < 0.05; Fig. S2D). Their samples also contained slightly fewer OTUs than those from lean individuals (BMI = <25), although this difference was not statistically significant (Mann-Whitney test; *P* > 0.05; Fig. 1C). The same trend toward lower diversity in overweight individuals was observed in the Montreal and Nunavut populations analyzed separately and also in a much larger data set of North Americans (Fig. 2). Within a given BMI bin, there was a slight (not statistically significant) tendency for individuals from Nunavut to have higher diversity than Montrealers (Fig. S3).

10.1128/mSphere.00297-16.4 FIG S3  Comparison of alpha diversity between Montreal and Nunavut, binned by BMI, using observed OTUs (A), Shannon’s diversity index (A), Simpson’s index (C), and Fisher’s alpha (D). While there is a tendency for greater diversity in Nunavut for a given BMI, the difference is not statistically significant (Mann-Whitney test; *P* > 0.05). OTUs were counted using the open-reference method (see Materials and Methods). Download FIG S3, PDF file, 0.1 MB. Copyright © 2017 Girard et al. 2017 Girard et al. This content is distributed under the terms of the Creative Commons Attribution 4.0 International license.

### The Inuit microbiome is broadly similar to the Western microbiome.

**FIG 3:**
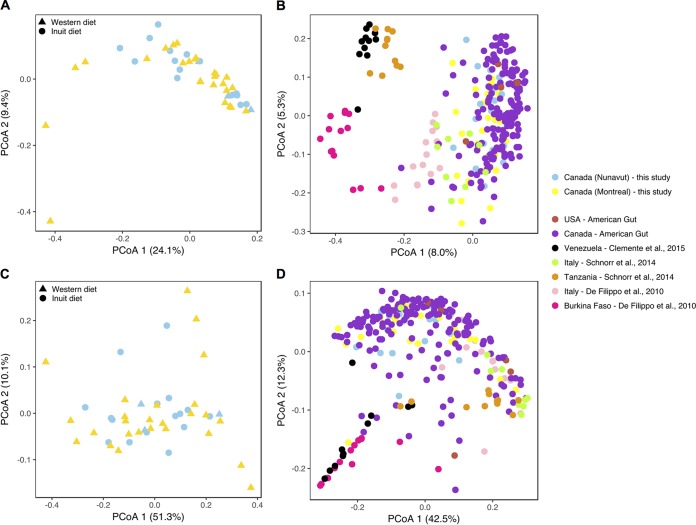
The Inuit microbiome has a community composition similar to that of the Western microbiome. (A and C) Montreal and Nunavut microbiomes cluster together, regardless of diet, based on principal coordinates analysis of unweighted (A) and weighted (C) UniFrac distances computed from open-reference OTUs (see Materials and Methods). Gap statistics analyses identified only one cluster, showing that the two populations overlap at the overall microbial community level. (See Fig. S4A and B for additional distance metrics.) (B and D) Montreal and Nunavut microbiomes cluster with other Western microbiomes sampled in other studies. Interstudy comparisons were performed with unweighted (B) and weighted (D) UniFrac distances computed from closed-reference OTU tables to limit interstudy variability. Binning samples by traditional agrarian/hunter-gatherer populations (Burkina Fason, Tanzania, Venezuela) and Western populations (United States, Italy, Montreal, Nunavut) explains 6.5% and 10.5% of the variation in the combined data sets (adonis; *P* < 0.001) for unweighted and weighted UniFrac data, respectively.

Previous comparisons of Western and traditional diets have also found significant differences in overall gut community composition, attributable to genetics, cultural practices, diet, or geography (8–12). In contrast, we found that the Montreal and Nunavut community compositions were similar and did not cluster according to geographic location or diet (Fig. 3A and C). This lack of clustering was observed regardless of the distance metric used to compare microbiomes (Fig. S4A and B). The Montreal cohort included two possible outliers (visible in the bottom-left quadrant of Fig. 3A) which also contained relatively few OTUs (two lowest points in Fig. 1A). However, these samples likely lack rare OTUs, as they cluster with other samples in the weighted UniFrac principal-coordinate analysis (PCoA) (Fig. 3C). The Montreal and Nunavut gut microbial community structures are therefore broadly similar, and both cohorts cluster near other Western populations and away from agrarian and hunter-gatherer groups from Burkina Faso, Tanzania, and Venezuela (Fig. 3B and D; adonis adjusted *R*^2^ = 6.5% and 10.5% for unweighted and weighted UniFrac, respectively; *P* < 0.001). While there is methodological bias involved in such a comparison (with study effects explaining 15.5% of variation in unweighted UniFrac and 17.1% in weighted UniFrac; adonis; *P* < 0.001), agrarian and hunter-gatherer populations clearly cluster apart from Western groups along PCoA axis 1, and Nunavut samples overlap with those from other Western populations. Genetic relatedness and ethnicity did not significantly explain clustering (Fig. S4C and D), which is consistent with a relatively minor effect of human genetics on overall microbiome composition, as has been observed in another indigenous population (21). We did not identify any other factor (age, gender, or BMI) that could explain the variation in gut microbial community structure (adonis; *P* > 0.05). We found that, consistent with broadly similar microbial communities, there were no significant differences between populations in stool short-chain fatty acid profiles (Fig. S5).

10.1128/mSphere.00297-16.5 FIG S4  Beta diversity analyses performed using Bray-Curtis analysis (A) and Jenson-Shannon divergence (B) reveal no clustering between populations or dietary types. See Text S1 for a description of the metrics. UniFrac distances of the gut microbial community show no clustering between members of the same family (C) or ethnic groups (D). Download FIG S4, PDF file, 0.1 MB. Copyright © 2017 Girard et al. 2017 Girard et al. This content is distributed under the terms of the Creative Commons Attribution 4.0 International license.

10.1128/mSphere.00297-16.6 FIG S5  Short-chain fatty acid (SCFA) content in stool samples according to geography (A) and to diet (B). (C) There were no significant differences between groups, and samples did not cluster together by geography based on similarity in SCFA profiles. Download FIG S5, PDF file, 0.05 MB. Copyright © 2017 Girard et al. 2017 Girard et al. This content is distributed under the terms of the Creative Commons Attribution 4.0 International license.

### Subtle differences distinguish the Inuit microbiome.

Despite the broad, community-level similarity of Nunavut and Montreal microbiomes, they could still differ in the relative abundances of certain microbial taxa. Such subtle differences might not affect overall community structure (Fig. 3A and B and S4), particularly if they involve relatively rare taxa or small changes in relative abundance. To identify microbial taxa that differed between cohorts, we compared all samples by geography (Montreal versus Nunavut) (Fig. S6A; Tables S1D and G), diet (Inuit diet versus Western diet) (Fig. S6B; Tables S1E and G), and BMI (BMI = <25 or >25) (Fig. S6C; Tables S1F and G). To disentangle diet from geography and genetics, we also compared the Inuit and Western diets among Nunavut participants only, who shared the same geographic location and were of mostly Inuit ancestry. Comparisons at higher taxonomic levels (phylum through family) were performed with linear discriminant analysis (to identify biomarkers, as defined by LEfSe) (22), and differentially abundant OTUs were identified using the negative binomial Wald test in DESeq2 (23, 24).

10.1128/mSphere.00297-16.7 FIG S6  (A to C) Diagram showing partitioning of samples and sample size compared across geography (A), diet (B), and BMI (C) data. (D) Venn diagram of OTUs identified by DESeq2 associated with geography (Nunavut or Montreal) and diet (Inuit diet or Western diet). Numbers in the Venn diagram represent numbers of OTUs associated with each category; numbers in overlapping sections represent numbers of OTUs associated with both categories; *n* is the total number of OTUs associated with a category. Most of the OTUs identified by DESeq2 as associated with Montreal were also associated with the Western diet (53.8%)—however, 1 OTU (*Prevotella copri* OTU 326482) was associated with Montrealers and the Inuit diet. Half of the OTUs associated with Nunavut were also more abundant in consumers of the Inuit diet (49.1%), while 4 OTUs were associated with the Western diet (*Eubacterium biforme* OTU 182483, an unclassified member of *Barnesiellaceae* family OTU 315846, an unclassified member of the RF39 family OTU 569244, and an unclassified member of the YS2 family OTU 269386). Raw data are presented in Tables S1D and E. Download FIG S6, PDF file, 0.1 MB. Copyright © 2017 Girard et al. 2017 Girard et al. This content is distributed under the terms of the Creative Commons Attribution 4.0 International license.

**FIG 4:**
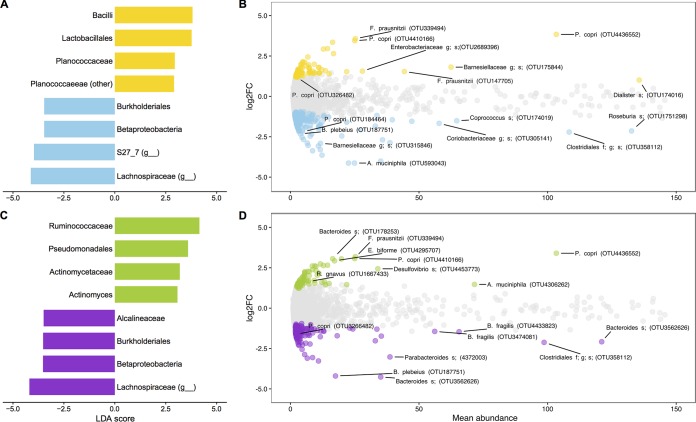
Differentially abundant OTUs and higher taxonomic units across geography and diet. (A and C) Linear discriminant analyses (LDA) using LEfSe were applied to identify biomarkers at higher taxonomic levels (down to the genus level). (B and D) Differentially abundant OTUs were identified using DESeq2 (see Materials and Methods). (A and B) Samples were compared across geographic regions (for Montreal, *n* = 26 [in yellow]; for Nunavut, *n* = 19 [in blue]) for LEfSe biomarkers (A) and differentially abundant OTUs (B) identified by DESeq2. (C and D) Samples were compared by diet (for the Western diet, *n* = 29 [in green]; for the Inuit diet, *n* = 19 [in purple]) for LEFSe biomarkers (C) and differentially abundant OTUs (D) identified by DESeq2. All associations had *P* values of <0.05 after correction for multiple tests. Only the data from the top four LEfSe biomarkers (LDA score of >2.5) for each category are presented here. For full LEfSe and DESeq2 results, see Tables S1D to G and Fig. S6A to C. The differentially abundant OTUs named as indicated in panels B and D focus on those discussed in the main text.

Comparing samples across geography, *Lactobacillales* and *Bacilli* were identified by LEfSe as the top two biomarkers for Montreal microbiomes (Fig. 4A; Table S1G), while *Ruminococcaceae* species were found to be associated with the Western diet (Fig. 4C; Table S1G). Bacteria were also identified as a biomarker for Montreal, because there were more unassigned reads in the Nunavut cohort (0.019% of reads per individual, compared to 0.015% in Montreal). DESeq2 analyses found Montreal samples to be enriched in two *Faecalibacterium prausnitzii* OTUs (OTU147702 and OTU339494) and three *Prevotella copri* OTUs (OTU326482, OTU4410166, and OTU4436552) (Fig. 4B; Table S1D). Meanwhile, the archaeal methanogen *Methanosphaera* was enriched in Nunavut (Table S1D). A different *P. copri* OTU (OTU184464), as well as an *Akkermansia muciniphila* OTU (OTU593043), was more abundant in Nunavut samples (Fig. 4B; Table S1D).

OTUs of *P. copri* and *A. muciniphila* were also identified as differentially abundant between Inuit and Western diets (Fig. 4D). The effects of diet and geography covaried substantially because all Montrealers consumed a Western diet and most participants from Nunavut consumed an Inuit diet. Consistent with this covariation, all biomarkers of the Inuit diet were also biomarkers of Nunavut (identified by asterisks in Table S1G). Of the 80 OTUs associated with Montreal, over half (*n* = 25) were also associated with the Western diet (Fig. S6D; Tables S1D and E). Only one of the 80 Montreal-associated OTUs was also associated with the Inuit diet. Of the 212 OTUs associated with Nunavut, 104 were significantly enriched in the Inuit diet. Four of the Nunavut OTUs, including one associated with BMI level of >25 (an unclassified OTU in the family *Barnesiellaceae*; Table S1F), were associated with the Western diet (Tables S1D and E). These results suggest that roughly 50% of geographic associations (43/80 and 104/212) are due to covariation between diet and geography.

### Low abundance and diversity of *Prevotella* in the Inuit diet.

To disentangle the effects of geography and diet, we attempted to identify biomarkers of diet within the Nunavut participants only. Probably due to the reduced power afforded by this limited sample size (*n* = 19), DESeq2 did not identify any OTUs associated with diet within Nunavut. However, LEfSe identified 14 taxonomic biomarkers of the Western diet in Nunavut; the top two biomarkers were the family *Prevotellaceae* and the genus *Prevotella* (Table S1G). *Prevotella* OTUs are present in both Montreal and Nunavut (Fig. 4; Table S1D), but there are more OTUs (7) associated with the Western diet than with the Inuit diet (1) (Table S1E). We therefore hypothesized that the Western diet harbored a greater diversity of *Prevotella* strains.

**FIG 5:**
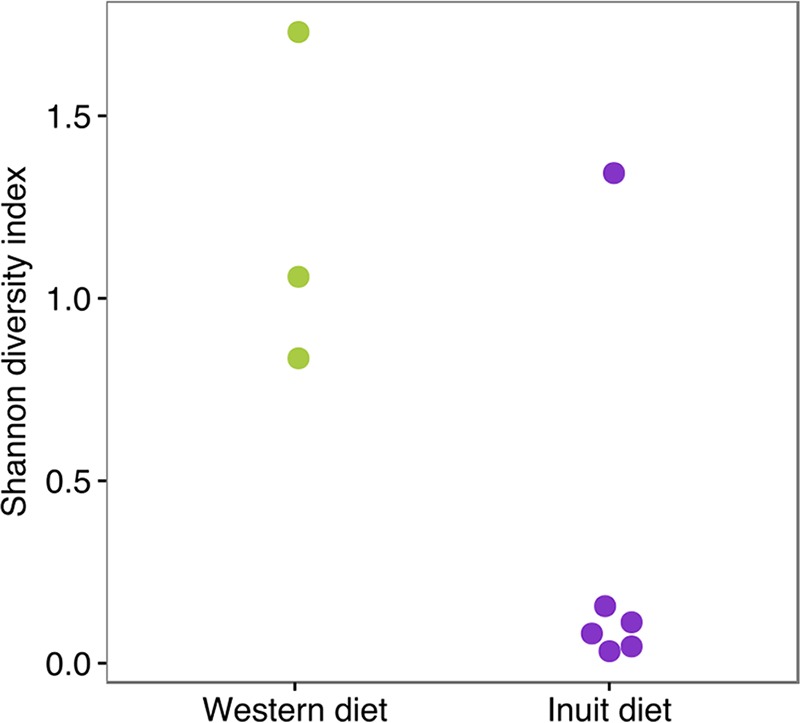
Inuit diet is associated with low *Prevotella* diversity. Nunavut participants consuming a Western diet had a significantly greater diversity of *Prevotella* strains (Shannon diversity of oligotypes) than those adhering to the Inuit diet (Mann-Whitney test; *P* < 0.05).

To test this hypothesis, we defined 48 strains of *Prevotella* using unsupervised oligotyping (Materials and Methods) and compared levels of strain diversity across diets within Nunavut (Fig. 5). We observed significantly lower *Prevotella* strain diversity in Nunavut participants consuming an Inuit diet than in those consuming a Western diet (Mann-Whitney test; *P* < 0.05). The five individuals with low diversity and an Inuit diet (Fig. 5) were unrelated and did not share a household, suggesting that *Prevotella* diversity was associated with diet rather than household transmission or human genetics. No effect of the Inuit diet on strain diversity was observed within either *Akkermansia* or *Bacteroides* (Mann-Whitney test; *P* > 0.05), suggesting that the effect is *Prevotella* specific. We divided Nunavut participants into four diet categories instead of two bins, and the data qualitatively confirmed that a more traditional Inuit diet was associated with reduced *Prevotella* strain diversity (Fig. S7).

10.1128/mSphere.00297-16.8 FIG S7  When diet was binned into 4 categories of increasing frequency of traditional food consumption (instead of 2, as in Fig. 5), we observed the same trend of low *Prevotella* diversity being associated with an increasingly traditional diet. The extreme Inuit diet (right; *n* = 4) is defined as individuals consuming traditional foods daily; the extreme Western diet (left; *n* = 1) is composed of individuals who never consumed traditional foods; intermediates (each *n* = 2) are composed, respectively, of individuals who rarely (less than weekly) or frequently (weekly) consume traditional foods (Tables S1A and C). Download FIG S7, PDF file, 0.02 MB. Copyright © 2017 Girard et al. 2017 Girard et al. This content is distributed under the terms of the Creative Commons Attribution 4.0 International license.

### Geographic and dietary associations of *Akkermansia* strains.

**FIG 6:**
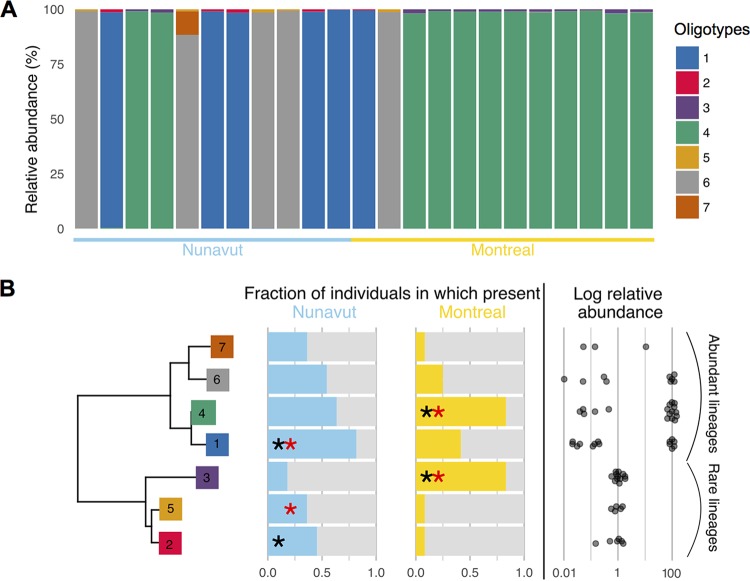
Two distinct *Akkermansia* lineages, each containing strains associated with geography and diet. (A) *Akkermansia* strains (oligotypes 1 to 7) across samples (individuals). Only individuals with at least 100 *Akkermansia* reads are included. Percentages are relative to the total number of *Akkermansia* reads in the individual. Most individuals were dominated by one single strain (representing >88% of reads) of 7 strains identified. (B) Neighbor-joining tree (left) of oligotype sequences, with the fraction of individuals in which the oligotype is present, and its mean abundance within individuals (right). Stars indicate significant associations of oligotypes with geography (Nunavut versus Montreal; black stars) and diet (Western versus Inuit diet; red stars) (LEfSe; *P* < 0.05 after correction for multiple tests; Table S1H).

OTUs within the *Akkermansia* genus, which is of interest with respect to human health and obesity (26), exhibited associations with both geography and diet (Fig. 4B and D; Tables S1D and E). We identified 8 strains of *Akkermansia* by unsupervised oligotyping, which we refined to 7 strains using supervised oligotyping. Two of these strains were associated with Montreal and the Western diet and three with Nunavut and/or the Inuit diet (Fig. 6A; Table S1H). We hypothesized that these strains might be phylogenetically grouped into two lineages—one corresponding to Montreal and one to Nunavut. However, inconsistent with this hypothesis, we constructed a phylogenetic tree of oligotypes and found two lineages (“Abundant” and “Rare”), each containing representatives in both Montreal and Nunavut (Fig. 6B). One lineage was always found at low abundance (~1% of *Akkermansia* oligotypes) and one at various levels of abundance, sometimes near 100% (Fig. 6).

## DISCUSSION

In previous studies of the gut microbiome, greater diversity has consistently been observed in agrarians and hunter-gatherers than in Western industrialized populations (8, 10–13). Our study in Nunavut thus provided a contrasting example of a modern traditional diet which is associated with diversity approximately equal to that associated with a Western diet (Fig. 1) and which clusters with those of other Western populations (Fig. 3). Those previous studies found large differences between Western and traditional microbiomes, despite the use of relatively small sample sizes (10–12). Using a similar sample size, we failed to detect large differences between Inuit and Western microbiomes. This suggests that modern Inuit and Western microbiomes are broadly similar, although additional sampling might detect subtle but significant differences in alpha and beta diversity. The broad similarity between Inuit and Western microbiomes could arise because even Inuit frequently consuming traditional foods are exposed also to at least some Western market food. This is consistent with an Inuit population nearing the end of their transition to a Western diet. More specifically, there are at least four factors that might explain the similarity of Inuit and Western microbiomes: BMI, fiber, meat consumption, and seasonality.

First, we observed that Inuit microbiomes tended to be slightly (but not significantly) more diverse than those of their southern Canadian counterparts, when binned by geography and BMI (Fig. 2; see also Fig. S3 in the supplemental material). The Inuit of the Canadian Arctic are undergoing a dietary transition, like many other indigenous populations, from a highly traditional to a Westernized diet (15, 17). This transition has occurred concurrently with increases in the prevalence of obesity in the Inuit (18, 27), which in turn has been linked with slightly (~2%) reduced gut microbiome alpha diversity (28). Consistently, we observed slightly reduced Shannon and Simpson diversity with higher BMI (Fig. S2D). The small effect of BMI could potentially mask a slightly elevated level of diversity in the Inuit microbiome. The ancestral state of the Inuit microbiome may thus have been of high diversity (like those of other traditional populations) (8, 10–13), as suggested by the higher diversity in Nunavut seen once the effects of BMI are removed (Fig. 2; Fig. S3). However, increasingly Westernized diets and increasing obesity may have led to reductions in diversity and to Western-like diversity in the Inuit (Fig. 1). Further studies using much larger sample sizes of Inuit will be needed to detect potentially small effects of traditional diet and BMI and their interaction on microbiome diversity.

Second, high gut microbiome diversity in traditional populations has frequently been associated with high fiber intake (10, 29), such as is typical in non-Western diets. Here, however, the difference in the levels of daily fiber intake between the Inuit (13.1 to 14.4 g for the Inuit of Nunavut [30]) and the average Western diet (15.1 g) (31) (both below daily recommended intakes) is negligible compared to the difference from agrarian populations with high-fiber diets (10, 11). Lack of diversity in the Inuit microbiome compared to other hunter-gatherers and agrarians could also be due to progressive loss of microbial diversity over generations of low-fiber traditional food consumption, typical of a Western diet (29).

Third, Inuit and Montrealers might both consume more meat than previously studied agrarians and hunter-gathers, possibly leading to similar levels of diversity and similar community structures in Inuit and Montreal microbiomes. It is known that shifts to extremely meat-based diets induce major changes in the gut microbiome (4, 32). However, these studies used extremely animal-only-based diets, while the Inuit diet includes at least some fiber (17) and many of our participants consumed some market food. Furthermore, previous studies (4, 32) followed a shift from a baseline to a meat-rich diet, while our Inuit participants have presumably been consuming a meat-rich diet throughout their whole lives. Therefore, some of the changes observed in previous studies could have been due to the drastic and rapid dietary changes rather than to the long-term effects of diet composition.

Fourth, seasonal microbiome variation has been observed in other human populations (10, 33) and might explain the similarity of the Montreal and Nunavut microbiomes. All sampling in this study was conducted in late July through early August, when most Inuit consume a mix of traditional and market food (20), which might be relatively similar to the Montreal diet. Other times of year, when more traditional food is consumed, might yield more distinct microbiomes. Taxa associated with the Inuit diet could thus be long-term dietary biomarkers, persisting even in periods of Western food consumption.

Despite the overall similarity between Inuit and Montreal microbiomes, they differed in the relative abundances of certain OTUs and higher taxonomic units, possibly linked to long-term dietary differences. For example, *F. prausnitzii*, which has been linked with consumption of citrus fruit (5), was overrepresented in Montreal, where—unlike the Arctic—citrus is readily available year-round. The enrichment of the *Lactobacillales* family in Montreal was also expected, because dairy products are widely consumed in Montreal but not in Nunavut. Finally, *Prevotella* has been previously associated with fiber-rich diets (10, 11) and was found as a biomarker of the Western diet within Nunavut. The association of *Prevotella* with the Western diet (in the entire cohort and specifically within Nunavut) is consistent with the greater amount of fiber in the Western diet than in the Inuit diet, even if the difference is slight compared to data from other agrarians and hunger-gatherers (10, 11, 18). Not only is the abundance of *Prevotella* greater in Inuit consuming a Western diet, the genus is more diverse in its richness and evenness of oligotypes (Fig. 5). It is possible that lower strain diversity in the Inuit diet group is due to the lower relative abundance of *Prevotella* in individuals consuming an Inuit diet (e.g., diversity exists, but strains are too rare to be detected). Some *Prevotella* OTUs (Fig. 4; see also Tables S1D and E in the supplemental material) and oligotypes (Table S1H) are associated with Western diet and geography and others with the Inuit diet. Together, these results suggest that populations consuming modestly different levels and types of fiber (e.g., diversity and abundance of fruits and vegetables) may differ in their relative abundances of different *Prevotella* strains. While *Prevotella* has been frequently associated with fiber-rich diets (10, 11, 34), the genus has also been linked to inflammation in the gut (35, 36). *Prevotella* strains vary from individual to individual (37) and strains might have contrasting associations with health state and diet (e.g., some may correlate with the presence of fiber while others may not) (38). Moreover, it is likely that factors other than fiber also contribute to shaping *Prevotella* diversity, in our samples and more generally.

In the case of *Prevotella*, we were able to disentangle the effects of diet and geography. However, the small sample size (*n* = 3) of Inuit consuming a Western diet prevented us from identifying differentially associated OTUs with DESeq2 and may have led to some false positives among the 14 LEfSe biomarkers. Larger sample sizes of Inuit consuming both traditional and Western diets will be needed to replicate and confirm these biomarkers.

In other cases, diet could not be clearly disentangled from geography, genetics, and lifestyle. For example, *Akkermansia* contained strains associated with either Montreal/Western diet or with Nunavut/Inuit diet (Fig. 6). Diet, geography, and lifestyle could all contribute to the distribution of these strains. The partitioning of these strains into two lineages also suggests an ancient divergence of “rare” and “abundant” lineages, followed by a more recent diversification into Montreal/Nunavut strains within each lineage, perhaps associated with environmental or dietary pressures. It remains to be seen if “rare” and “abundant” *Akkermansia* strains represent true monophyletic groups, if they are a general feature of other human microbiomes, and whether there are any ecological differences between them or between Montreal/Nunavut strains.

In summary, the Inuit harbor a diversity of gut microbes that is not strikingly different from that of their urbanized, Westernized counterparts. This may not reflect the ancestral Inuit microbiome: indeed, dietary transition and Westernization, as well as the increasing prevalence of obesity, may have reduced diversity and changed the composition of the Inuit microbiome over time. The modern Inuit microbiome resembles that of southern Canadians and other Western populations. We did, however, pinpoint subtle differences in the composition of the Inuit and Western gut microbiomes which may be due to contrasting diets. Like other native populations (21), the Inuit have a unique set of health risks, many of which could be modulated by the microbiome. Although investigation of health risks was not the goal of our study, we have presented a snapshot of an Inuit microbiome in transition, providing a foundation for future studies of how the microbiome changes over time and how it interacts with diet and human genetics to affect health and disease.

## MATERIALS AND METHODS

### Participant enrollment and sample collection.

We recruited 26 volunteers from the community of Resolute Bay, Nunavut (representing approximately 18% of the local adult population), a small hamlet where 95% of the population is Inuit (see Fig. S1A and S1B in the supplemental material) (39). Three individuals of European descent living in Resolute Bay were included in the study. We also recruited 33 residents of Montreal, Canada, most of whom were working or studying at a university and all of whom were of European descent. Stool samples were collected from July to September 2014 from healthy participants who had not taken antibiotics in the previous 3 months. Details on volunteer characteristics are presented in Table S1B in the supplemental material.

All volunteers gave written informed consent after the objectives and potential outcomes of the study were explained to them. Participants completed dietary habit questionnaires, evaluating their typical diet over the course of a year (Table S1C). Dietary information was compiled according to frequency of traditional Inuit food consumption, spanning a range from an entirely Western diet to a highly traditional Inuit diet. For all subsequent analyses, dietary information was broken down into two categories: Inuit diet (individuals who consumed traditional Inuit food at least twice a week) and Western diet (individuals who only occasionally or never ate traditional Inuit food). The Western diet category included individuals from both Montreal and Nunavut who consumed traditional Inuit food infrequently or never. All work was approved by the Université de Montréal ethics review board for arts and sciences (CERFAS; certificate no. 2013-14-022-D). Permission for this work was granted by the Nunavut Research Institute (licenses no. 2 040 13N-A and 02 046 14N-A) and by the Hunters and Trappers Association and Hamlet of Resolute Bay.

Participants wore sterile gloves while collecting stool samples into sterile specimen cups. In Nunavut, samples were kept outside (at temperatures of <4°C) for a maximum of 12 h before being collected by a sampling team and frozen at −80°C. In Montreal, samples were immediately frozen at −20°C before being collected by a sampling team and frozen at −80°C.

### DNA extraction, library preparation, and sequencing.

DNA was extracted from stool samples using a PowerSoil DNA isolation kit (Mo Bio Laboratories, Inc.) (4). Library preparation was done using a two-step PCR method to amplify the v4 region of the 16S rRNA gene (see Text S1 in the supplemental material). During the first step of PCR, primers PE16S_V4_U515_F (5′ ACACG ACGCT CTTCC GATCT YRYRG TGCCA GCMGC CGCGG TAA 3′) and PE16S_V4_E786_R (5′-CGGCA TTCCT GCTGA ACCGC TCTTC CGATC TGGAC TACHV GGGTW TCTAA T 3′) were used to target and amplify the v4 region, as well as to add second-step priming sites (40, 41). Library size was confirmed at approximately 440 bp with a Qiaxcel Advanced system (Qiagen). Libraries were quantified with a Qubit v.2.0 fluorometer (Life Technologies, Inc.) and were pooled and denatured following the Illumina protocol. Paired-end sequencing (2 × 250 bp) was performed using MiSeq reagent kit V2 (Illumina) and a MiSeq sequencer (Illumina). All sequencing was done in a single run, with a Q score greater than Q30 for 93.1% of reads, and a cluster density of 856 ± 12 K mm^−2^.

### OTU picking and data processing.

We obtained 26 samples from our Nunavut participants, 19 of which were successfully sequenced. Meanwhile, of the 33 Montreal samples collected, 26 were successfully sequenced (>5,000 raw reads). The sequencing data were analyzed using QIIME (version 1.8.0) (42). Paired-end reads were concatenated using the join_paired_ends.py script and default parameters. Libraries were demultiplexed with the split_libraries_fastq.py script according to barcode identification. Chimeric sequences were identified using the usearch 61 method with the identify_chimeric_seqs.py script and were removed using the filter_fasta.py script. Sequencing produced a total of 6,345,335 reads and an average of 141,007 ± 54,899 reads per sample. Each sample was rarefied to 50,000 reads for subsequent analyses (unless otherwise indicated, e.g., for DESeq2 analyses). Open-reference operational taxonomic unit (OTU) picking was performed in QIIME (pick_open_reference_otus.py script) at a 97% identity level using Greengenes version 13_8 with a prefiltering step to remove non-16S sequences (percent identity, <60%) (43). OTUs with fewer than 10 observations across all samples were filtered from the OTU table (filter_otus_from_otu_table.py script). This left us with a final data set of 45 samples and 9,581 OTUs.

### Data analyses.

Alpha diversity was computed using the Phyloseq package (25) in R (44) with several metrics: observed OTUs; Chao1-estimated OTUs; and Shannon, Simpson, and Fisher diversity indices. The levels of OTU diversity in our data set were compared to those determined for 1,000 randomly selected American Gut project samples (ftp://ftp.microbio.me/AmericanGut/latest) (see Text S1 for data filtering). For this comparison, we performed closed-reference OTU picking (pick_closed_reference_otus.py) at 97% identity, eliminated OTUs with fewer than 10 observations across all samples (filter_otus_from_otu_table.py), and rarefied samples to 10,000 reads (single_rarefaction.py).

Beta diversity analyses were performed on weighted and unweighted UniFrac and Bray-Curtis distances, as well as Jenson-Shannon divergence, and were then visualized using PCoA and the ggplot2 R package (45). Sample groups were compared by analysis of variance using a permutation test with pseudo-*F* ratios with the adonis() function (R vegan package) (46). Clusters of samples were analyzed using the gap statistic, which estimates the number of clusters (groups) in a data set (47). We compared our data to 16S sequences from De Filippo et al. (EBI: project “ERP000133”), Schnorr et al. (MG-RAST: project ID “7058”), and Clemente et al. (EBI: projects “ERA387449” and “ERP008799”) and publicly available sequences from the American Gut Project, filtered as described in Text S1 (8, 10–12). To minimize methodological differences among studies, we performed closed-reference OTU picking, and OTUs with fewer than 10 observations across all samples were removed. Samples were then rarefied to 1,000 reads per sample and compared using unweighted and weighted UniFrac distances.

We performed linear discriminant analyses (LDAs) using LEfSe to identify microbial taxa (biomarkers, at all taxonomic levels, down to the genus level) that characterize the differences between groups of samples. The alpha value for Kruskal-Wallis and Wilcoxon tests was set at 0.05, the logarithmic LDA score threshold was 2.0, and per-sample normalization of sum values was applied (LEfSe default parameters). These biomarkers are microbial taxa that differ in abundance between groups, as identified by a Wilcoxon rank-sum test. The effect size of each biomarker was then estimated by determining an LDA score (22). When LEfSe analyses were initially performed on our data set, the domain *Bacteria* emerged as a biomarker for Montreal. We determined that this was due to unassigned reads being more extensively associated with the Nunavut cohort. However, while the difference between populations was large enough to impact LEfSE analyses, unassigned reads accounted for a very small proportion of reads (average of 0.02% of reads per sample). We removed unassigned reads, and *Bacteria* no longer represented a biomarker. All other LEfSE results remained identical and are reported here.

To investigate differences at a finer taxonomic level (the OTU level), we performed differential abundance analyses on unrarefied and filtered (minimum of 10 observations across all samples) OTU tables using DESeq2 (23, 24). Only taxa found to be significant (*P* < 0.05 after multiple-hypothesis testing) were reported.

To define strains within certain genera of interest, we used unsupervised oligotyping (also known as Minimum Entropy Decomposition) (MED) (version 0.1-alpha; http://oligotyping.org/MED) (48). By using the Shannon entropy, MED decomposes the data set to find “MED nodes” that explain the maximum entropy. To filter noise, we removed MED nodes for which the most abundant unique sequence was represented by fewer than 100 reads (-M 100). We found 8 MED nodes (strains) within *Akkermansia*, 48 within *Prevotella*, and 256 within *Bacteroides*. We calculated the Shannon diversity of these strains as described above. We excluded individuals (samples) with fewer than 100 reads within the genus of interest. For genera with relatively few MED nodes (*Akkermansia* and *Prevotella*), we were able to confirm the results with supervised oligotyping (http://merenlab.org/software/oligotyping/) (49, 50). Using oligotyping v1.4, we identified 45 *Akkermansia* oligotypes and 7 *Prevotella* oligotypes. We found that the minimal numbers of nucleotide positions explaining the diversity within these genera were, respectively, 36 and 14 high-entropy positions. Figure S8 shows the distribution of entropy along the *Akkermensia* and *Prevotella* reads and positions. In order to minimize the impact of noise, we used parameters that removed any oligotypes with a frequency smaller than 100 modified (-M 100), and we eliminated oligotypes that appeared in fewer than three samples (-s 3). These filters removed 1.63% of *Prevotella* reads and 7.95% of *Akkermansia* reads. We used LEfSe as described above to identify oligotypes (strains) associated with diet and/or geography (Table S1H). On the basis of the 11 high-entropy positions in the *Akkermansia* alignment, we constructed a neighbor-joining tree of the 7 oligotypes (strains).

10.1128/mSphere.00297-16.10 FIG S8  Shannon entropy at each position of the 16S alignment for *Prevotella* (A) and *Akkermansia* (B) oligotypes. Download FIG S8, PDF file, 0.9 MB. Copyright © 2017 Girard et al. 2017 Girard et al. This content is distributed under the terms of the Creative Commons Attribution 4.0 International license.

### Short-chain fatty-acid analysis.

Short-chain fatty acids from stool samples were quantified by gas chromatography coupled with a flame ionization detector (GC-FID). For details of the methods, see Text S1.

### Data availability.

Raw 16S rRNA gene sequences have been deposited in Qiita (http://qiita.microbio.me/) under study ID 10439 and are available on GitHub (https://github.com/cgir/16S_inuitgut).
