# Supplementary figures and images for: Gut Microbiome of the Canadian Arctic Inuit
Source: mSphere. 2017 Jan 4;2(1):e00297-16. doi: 10.1128/mSphere.00297-16 (PMC5214747; doi:10.1128/mSphere.00297-16)

**A**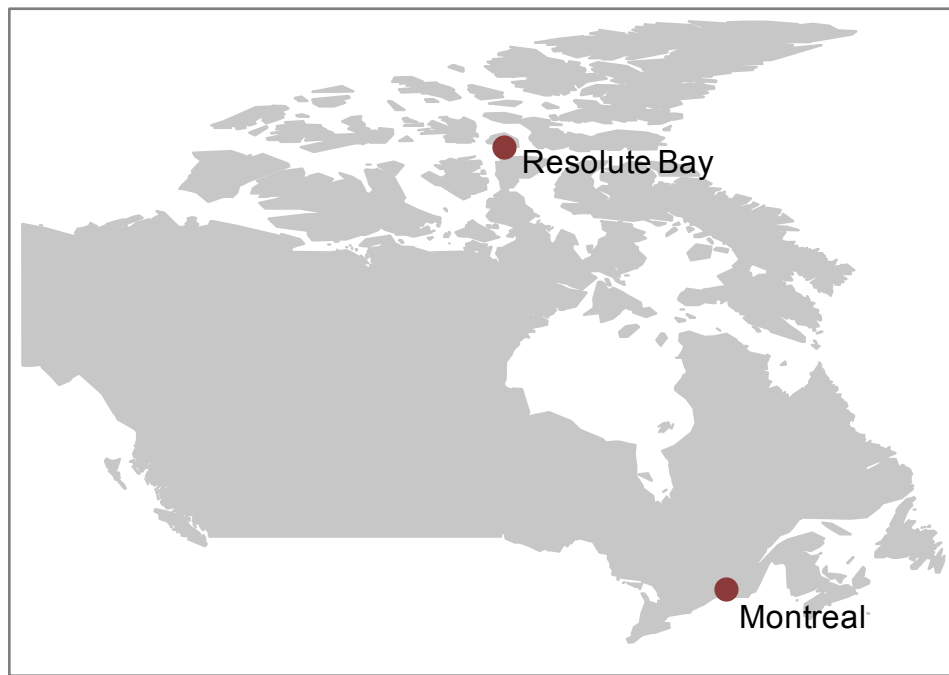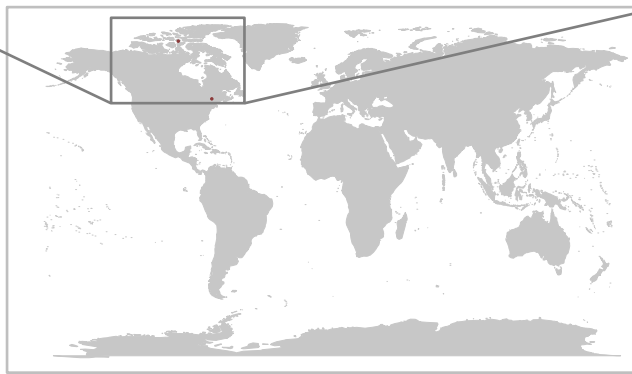**B**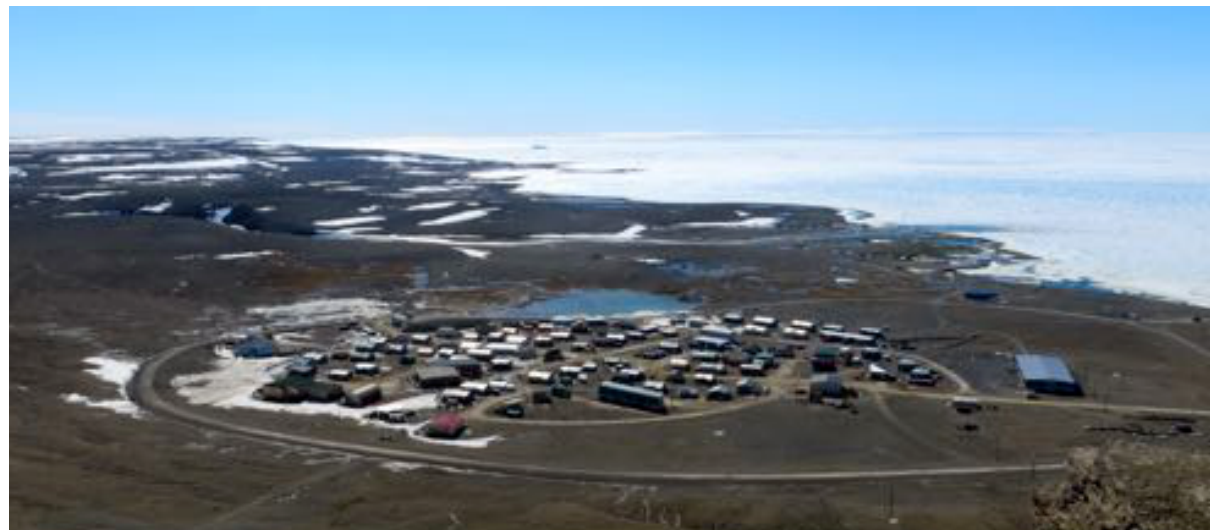**C**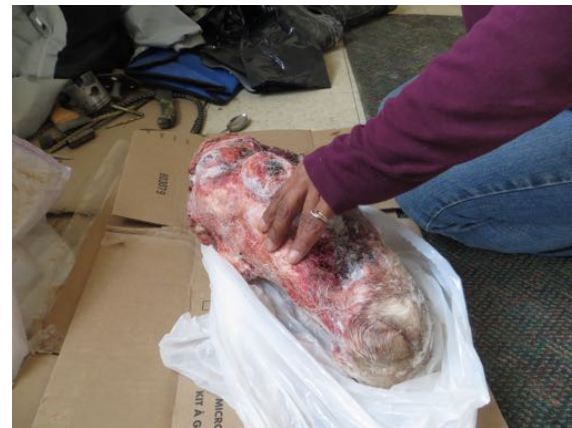**D**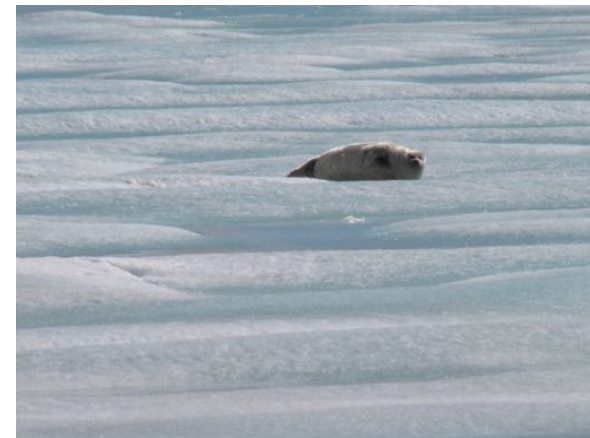

Supplement: FIG S1 [file sph001172219sf2.pdf]

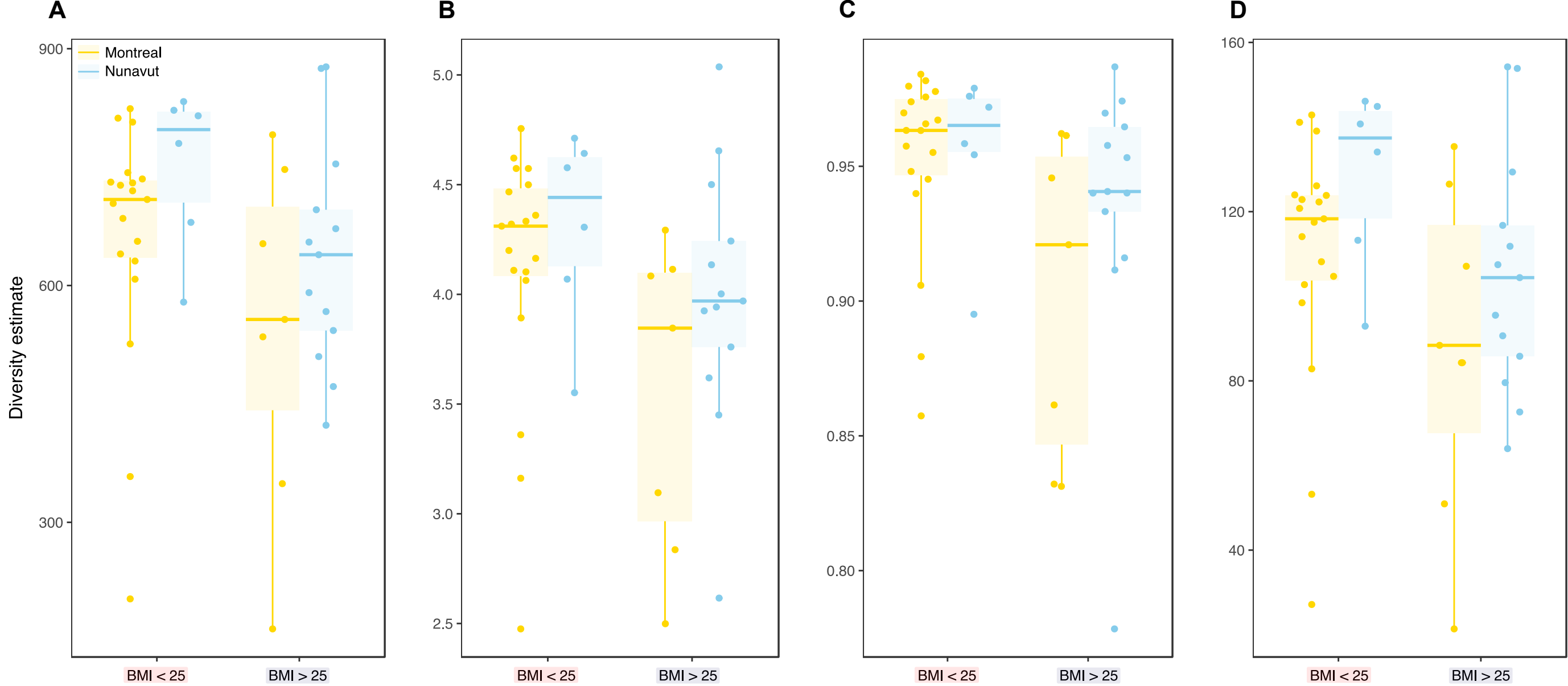

Supplement: FIG S3 [file sph001172219sf4.pdf]

**A**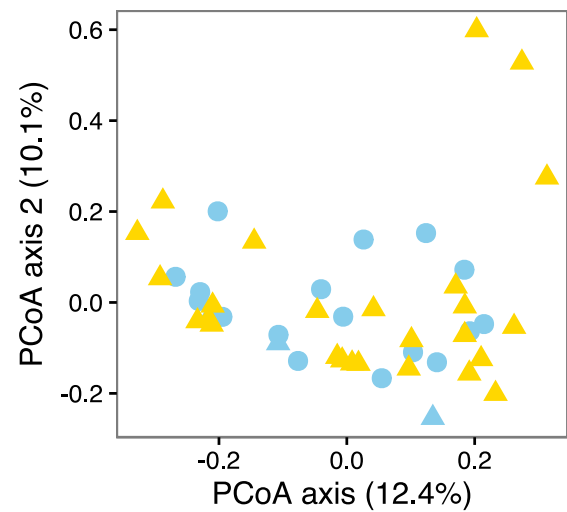**B**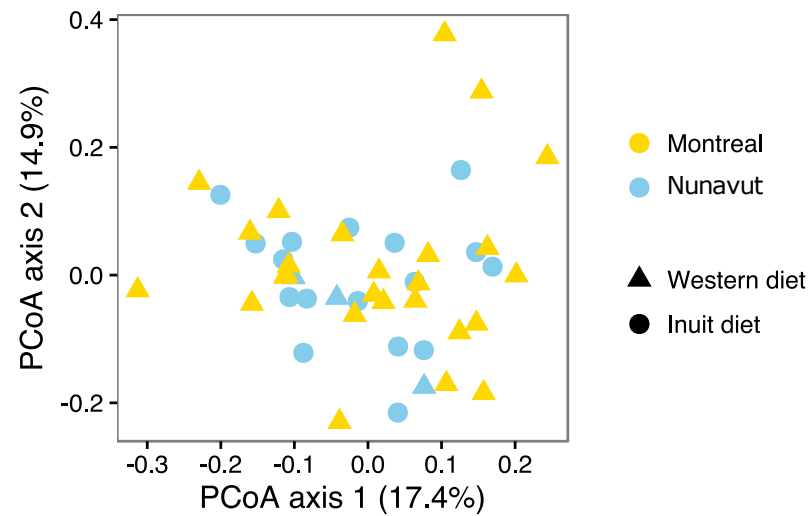**C**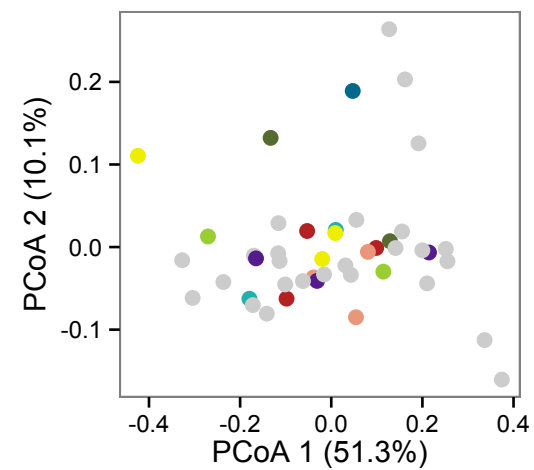**D**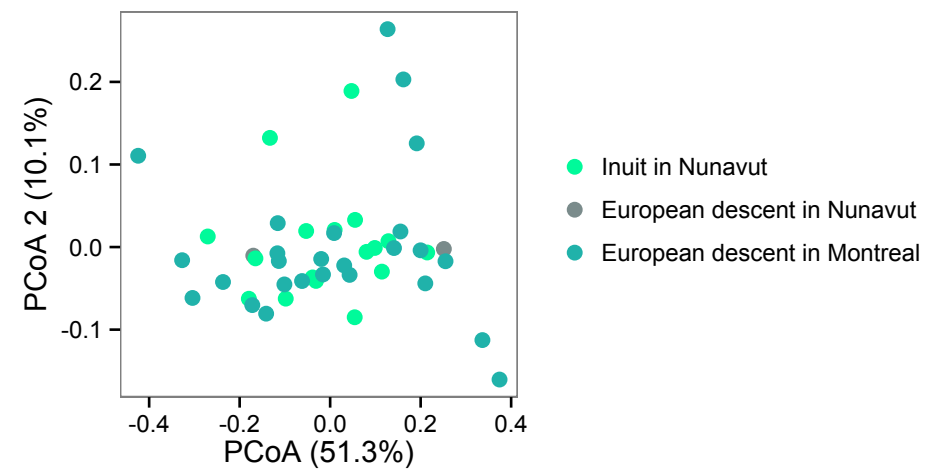

Supplement: FIG S4 [file sph001172219sf5.pdf]

**A**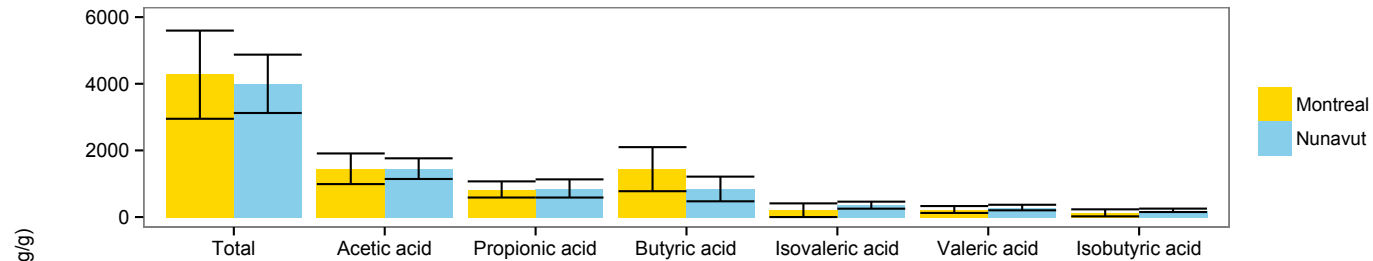**B**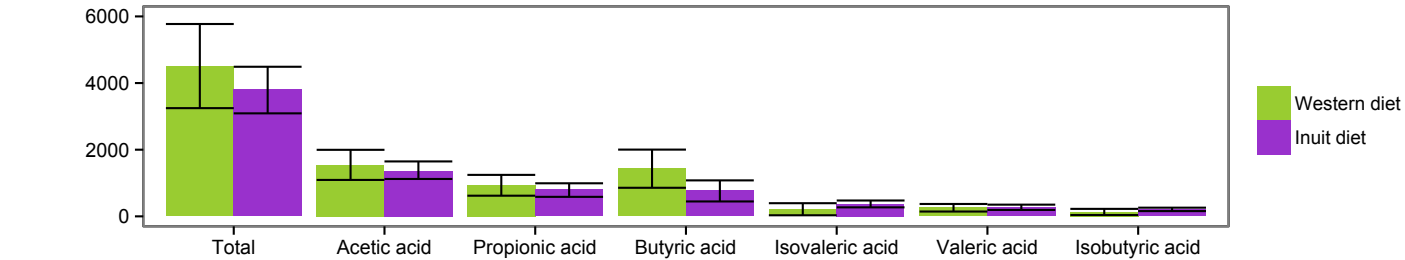**C**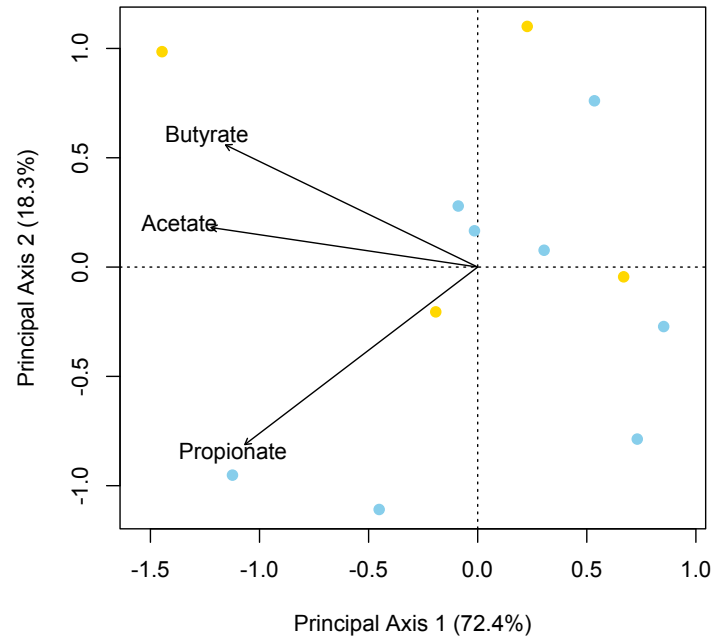

Supplement: FIG S5 [file sph001172219sf6.pdf]

COMPARISONS

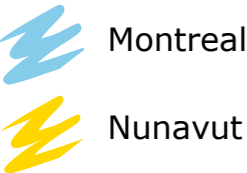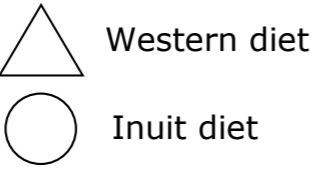

----- BMI > 25

A. Geography

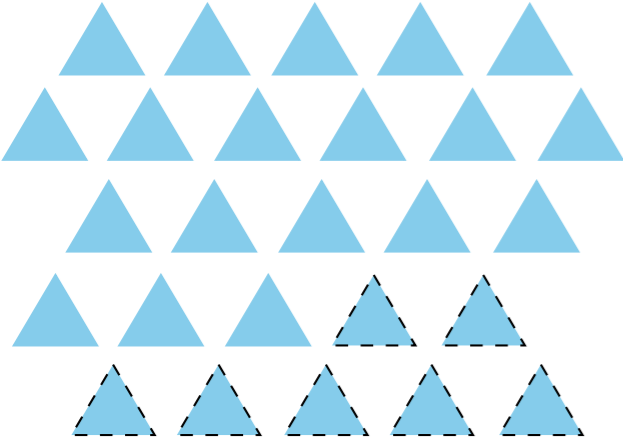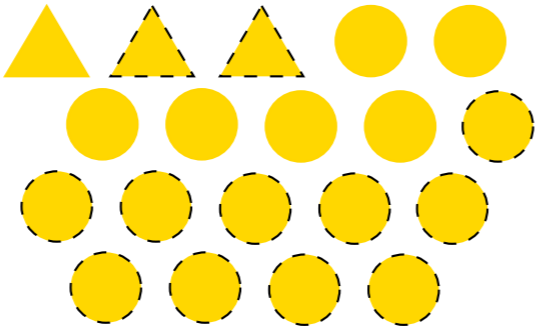

B. Diet

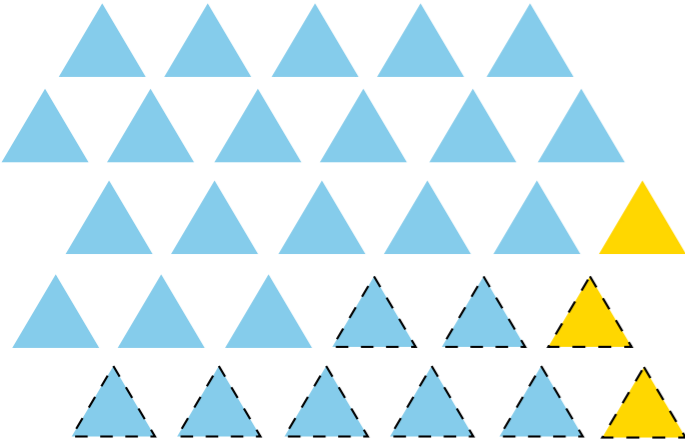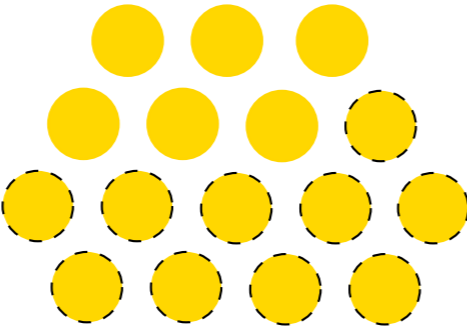

C. BMI

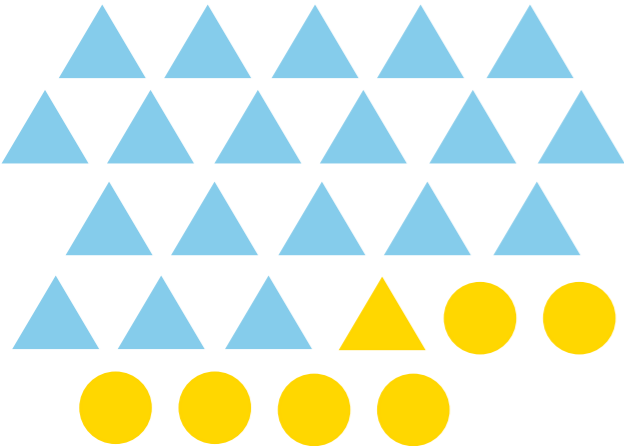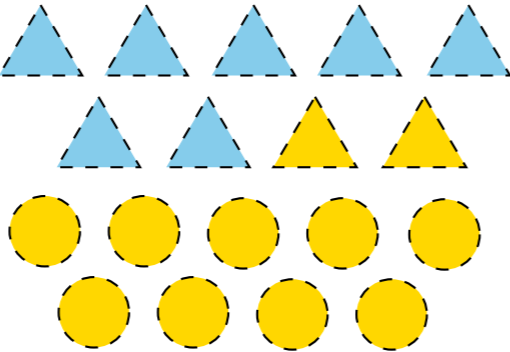

D. OTU associations

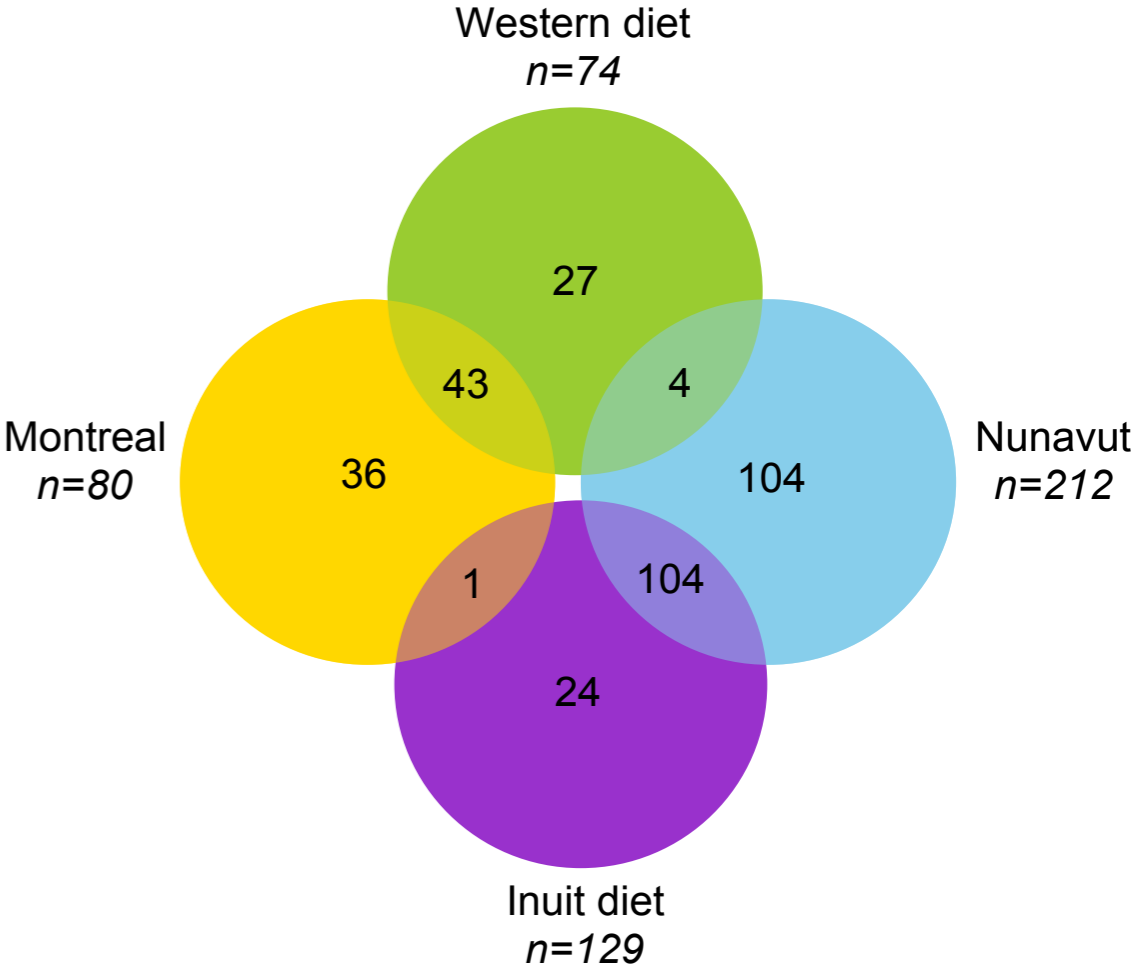

Supplement: FIG S6 [file sph001172219sf7.pdf]

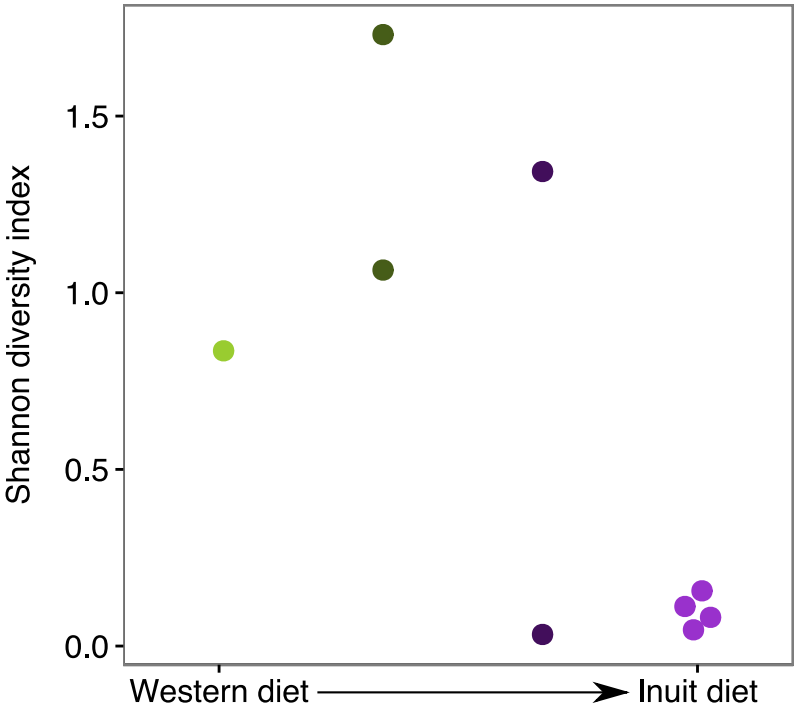

Supplement: FIG S7 [file sph001172219sf8.pdf]

**A**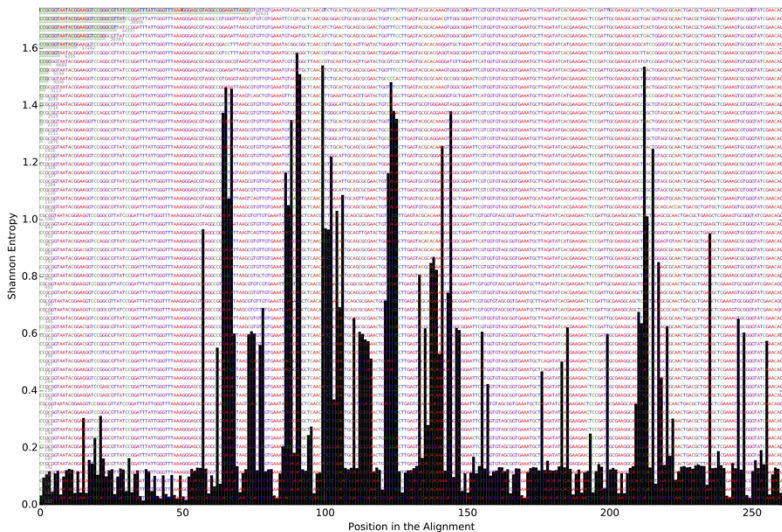**B**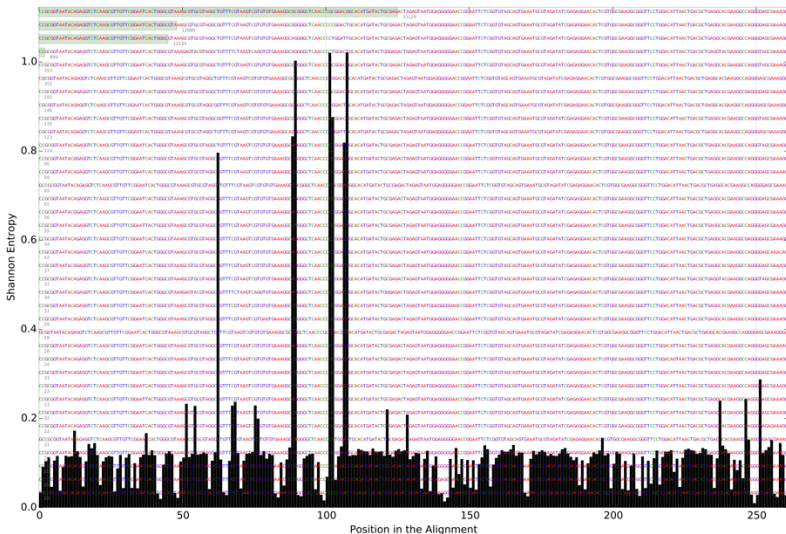

Supplement: FIG S8 [file sph001172219sf9.pdf]
