# Supplementary material for: Gut Microbiome of the Canadian Arctic Inuit
Source: mSphere. 2017 Jan 4;2(1):e00297-16. doi: 10.1128/mSphere.00297-16 (PMC5214747; doi:10.1128/mSphere.00297-16)
Supplement: FIG S2 [file sph001172219sf3.pdf]

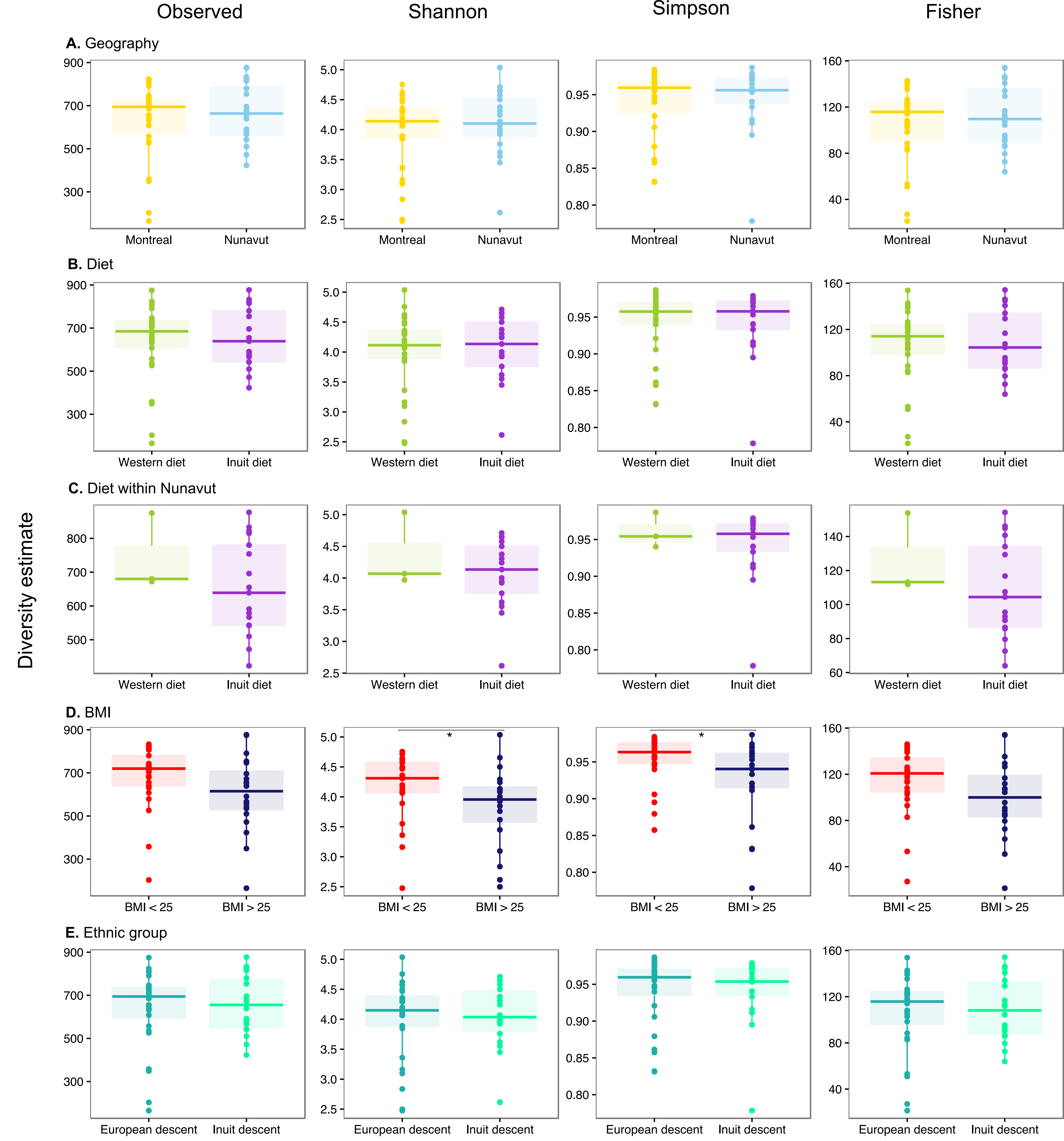
Observed
Shannon
Simpson
Fisher
A. Geography
Montreal
Nunavut
B. Diet
Western diet
Inuit diet
C. Diet within Nunavut
Western diet
Inuit diet
D. BMI
BMI < 25
BMI > 25
*
E. Ethnic group
European descent
Inuit descent
Diversity estimate
