# Supplementary material for: Gut Microbiome of the Canadian Arctic Inuit
Source: mSphere. 2017 Jan 4;2(1):e00297-16. doi: 10.1128/mSphere.00297-16 (PMC5214747; doi:10.1128/mSphere.00297-16)
Supplement: TEXT S1 [file sph001172219s1.docx]

Supplementary Materials and Methods

## *Participant enrollment and sample collection*

We recruited 26 volunteers from the community of Resolute Bay, Nunavut (representing approximately 18% of the local adult population), a small hamlet where 95% of the population is Inuit (Fig. S1A and S1B) (1). Three individuals of European descent living in Resolute Bay were included in the study. We also recruited 33 residents of Montreal (Canada) mostly working or studying at a university, and all of European descent. Stool samples were collected from July-September 2014 from healthy participants who had not taken antibiotics in the previous 3 months. Details on volunteer characteristics are presented in Table S1B.

All volunteers gave written informed consent after the objectives and potential outcomes of the study were explained to them. Participants completed dietary habit questionnaires, evaluating their typical diet over the course of a year (Table S1C). Dietary information was compiled according to frequency of traditional Inuit food consumption, spanning a range from an entirely Western diet to a highly traditional Inuit diet. For all subsequent analyses, dietary information was broken down into two categories: Inuit (individuals who consumed traditional Inuit food at least twice a week) or Western diet (individuals who occasionally or never ate traditional Inuit food). The Western diet category included individuals from both Montreal and Nunavut who consumed traditional Inuit food infrequently or never. All work was approved by the Université de Montréal ethics review board for arts and sciences (CERFAS, certificate #2013-14-022-D). Permission for this work was granted by the Nunavut Research Institute (licenses #02 040 13N-A and #02 046 14N-A), by the Hunters & Trappers Association and Hamlet of Resolute Bay.

Participants wore sterile gloves while collecting stool samples into sterile specimen cups. In Resolute Bay, samples were kept outside (temperatures < 4 °C) for a maximum of 12 hours before being collected by a sampling team and frozen at -80 °C. In Montreal, samples were immediately frozen at -20 °C before being collected by a sampling team and frozen at -80 °C.

## *DNA extraction, library preparation and sequencing*

DNA was extracted from stool samples using the PowerSoil DNA Isolation Kit (MO BIO Laboratories, Inc.) (2). Library preparation was done using a two-step PCR method to amplify the V4 region of the 16S rRNA gene. During the first step PCR, primers PE16S_V4_U515_F (5′ ACACG ACGCT CTTCC GATCT YRYRG TGCCA GCMGC CGCGG TAA-3′) and PE16S_V4_E786_R (5′-CGGCA TTCCT GCTGA ACCGC TCTTC CGATC TGGAC TACHV GGGTW TCTAA T 3′) were used to target and amplify the V4 region, as well as to add second-step priming sites (3, 4). The first step PCR reaction was performed as follows: 0.5 units of Phusion polymerase (New England BioLabs) with 1X High Fidelity buffer, 0.5 μL dNTPs, 2.5 μL of 3 μM PE16S_V4_U515_F and PE_16S_V4_E786_R primers and 5 ng of DNA were combined in a 25 μL reaction. PCR reactions were performed under the following conditions: initialization step at 98 °C for 30 seconds, denaturation at 98 °C for 25 seconds, annealing at 54°C for 40 seconds, extension at 72 °C for 30 seconds and final elongation at 72 °C for 2 minutes, over 20 cycles. Each sample was prepared in quadruplicate, with replicates pooled to a final volume of 100 μL and purified by Agencourt AMPure XP purification (Beckman Coulter), following the manufacturer’s protocol.

During the second-step PCR amplification, 9-bp barcodes for sample identification and Illumina adapter sequences were added to step one products, using second step primers PE-III-PCR-F (5′-AATGA TACGG CGACC ACCGA GATCT ACACT CTTTC CCTAC ACGAC GCTCT TCCGA TCT 3′) and PE-III-PCR-001-096 (5′-CAAGC AGAAG ACGGC ATACG AGATN NNNNN NNNCG GTCTC GGCAT TCCTG CTGAA CCGCT CTTCC GATCT 3′), where Ns represent the unique barcode (3). The second step PCR reaction was performed as follows: 5 units of Phusion polymerase with 1X High Fidelity buffer, 0.5 μL of dNTPs, 3.3 μL of 3 μM PE-PCR-III-F and PE-PCR-III-001-096 and 4 μL of step one product were combined in a 25 μL reaction. PCR reactions were performed under the following conditions: initialization step at 98 °C for 30 seconds, denaturation at 98 °C for 30 seconds, annealing at 83°C for 30 seconds, extension at 72 °C for 30 seconds and final elongation at 72 °C for 2 minutes, over 7 cycles. Each sample was prepared in quadruplicate, with replicates pooled to a final volume of 100 μL and purified by Agencourt AMPure XP purification (Beckman Coulter), following the manufacturer’s protocol.

Library size was confirmed at approximately 440 bp with a Qiaxcel Advanced System (QIAGEN). Libraries were quantified with a Qubit v.2.0 fluorometer (Life Technologies), pooled and denatured following the Illumina protocol. Paired end sequencing (2 x 250 bp) was performed using MiSeq reagent Kit V2 (Illumina) on the MiSeq (Illumina). All sequencing was done in a single run, with Q score was greater than Q30 for 93.1% of reads, and cluster density was of 856 ± 12 K mm^-2^.

## *OTU picking and data processing*

We obtained 26 samples from our Nunavut participants, 19 of which were successfully sequenced (>5000 raw reads per sample). Meanwhile, of the 33 Montreal samples collected, 26 were sequenced (>5000 raw reads). The sequencing data was analyzed using QIIME (version 1.8.0) (5). Paired-end reads were concatenated using the join_paired_ends.py script. Libraries were demultiplexed with the split_libraries_fastq.py script according to barcode identification. Chimeric sequences were identified using the usearch 61 method with the identify_chimeric_seqs.py script, and removed with the filter_fasta.py script. Sequencing produced a total of 6,345,335 reads and an average of 141,007 ± 54,899 reads per sample. Each sample was rarefied to 50,000 reads for subsequent analyses (unless otherwise indicated, *e.g.* for DESeq2 analyses). Open-reference operational taxonomic unit (OTU) picking was performed in QIIME (pick_open_references.py script) at a 97% identity level, with a pre-filtering step to remove non-16S sequences (percent identity < 60%) (6). OTUs with fewer than 10 observations across all samples were filtered from the OTU table (filter_otus_from_otu_table.py script). This left us with a final data set of 45 samples and 9,581 OTUs.

## *Data analyses*

Alpha diversity was computed using the Phyloseq package (7) in R (8) with several metrics: observed OTUs, Chao1 estimated OTUs, Shannon, Simpson and Fisher diversity indices. The observed species metric counts the number of distinct OTUs, chao1 estimates richness, while Shannon’s diversity index, Simpson’s index and Fisher’s alpha include measures of richness and community evenness. We compared our alpha diversity results to publicly available sequences from the American Gut project ([ftp://ftp.microbio.me/AmericanGut/latest)](https://github.com/biocore/American-Gut)), randomly selecting 1,000 ‘healthy’ samples from this dataset (‘healthy’ as described here, with the exception of a BMI range of 18.5-45, to match that of our Montreal/Nunavut samples: <ftp://ftp.microbio.me/AmericanGut/paper_healthy_subset.txt)>. Briefly, we performed chimera filtering (identify_chimeric_seqs.py) in both datasets, and American Gut sequences were filtered for ‘bloom’ sequences attributable to transport by mail using SortMeRNA 2.0, as described here: <http://americangut.org/removing-blooming-bacterial-sequences-from-american-gut-data/>. To minimize methodological differences among studies, we then performed closed-reference OTU picking (pick_closed_reference_otus.py script) at 97% identity, and filtered out OTUs with less than 10 observations across all samples. Each sample was rarefied to 10,000 reads, and we compared observed OTUs across geography in both datasets.

Beta diversity analyses were performed on weighted and unweighted UniFrac and Bray-Curtis distances, as well as Jenson-Shannon divergence, then visualized with PCoA using the ggplot2 R package (9). Jensen-Shannon divergence (JSD) and Bray-Curtis measure similarity across two populations, while UniFrac does the same but includes information on the relatedness of OTUs by using phylogenetic distances (10).

Sample groups were compared with the adonis() function (analysis of variance using a permutation test with pseudo-*F* ratios, R vegan package) (11). Clusters of samples were analyzed using the gap statistic, which estimates the number of clusters (groups) in a dataset (12). We compared our data to 16S sequences from De Filippo et al. (EBI: project ‘ERP000133’), Schnorr et al. (MG-RAST: project ID ‘7058’) and Clemente et al. (EBI: projects ‘ERA387449’ and ‘ERP008799’), and 200 randomly selected ‘healthy’ American Gut Project samples, filtered as described above ([ftp://ftp.microbio.me/AmericanGut/latest)](https://github.com/biocore/American-Gut)) (13–15). We performed closed-reference OTU picking (pick_closed_reference_otus.py script) at 97% identity, and OTUs with less than 10 observations across all samples were filtered out. Reads were then rarefied to 1,000 reads per sample, and plotted using weighted and unweighted UniFrac.

We performed linear discriminant analyses (LDA) using LEfSe to identify microbial taxa (biomarkers, at all taxonomic levels, down to the genus level) that characterize the difference between groups of samples. These biomarkers are microbial taxa that differ in abundance between groups, as identified by a Wilcoxon rank-sum test. The effect size of each biomarker is then estimated by an LDA score (16). To investigate differences at finer taxonomic levels (OTU level), we performed differential abundance analyses on unrarefied, filtered (minimum of 10 observations across all samples) OTU tables using DESeq2 (17). Only taxa found as significant (*P* < 0.05 after multiple hypothesis testing) were reported.

To define strains within certain genera of interest, we used unsupervised oligotyping (also known as Minimum Entropy Decomposition, MED version 0.1-alpha; <http://oligotyping.org/MED)> (18). By using the Shannon entropy, MED decomposes the dataset to find “MED nodes” that explain the maximum entropy. To filter noise, we removed MED nodes for which the most abundant unique sequence was represented by fewer than 100 reads (-M 100). We found eight MED nodes (strains) within *Akkermanisa,* 48 within *Prevotella*, and 256 within *Bacteroides*. We calculated the Shannon diversity of these strains as described above. We excluded individuals (samples) with fewer than 100 reads within the genus of interest. For genera with relatively few MED nodes (*Akkermansia* and *Prevotella*), we were able to confirm the results with supervised oligotyping (<http://oligotyping.org)> (19, 20). Using oligotyping v1.4, We identified 45 *Akkermansia* oligotypes and 7 *Prevotella* oligotypes. We found that the minimal number of nucleotide positions explaining the diversity within these genera was respectively 36 and 14 high-entropy positions. Supplementary Fig. S8 shows the distribution of entropy along the *Akkermensia* and *Prevotella* reads and positions. In order to minimize the impact of noise, we used parameters that removed any oligotypes with a frequency smaller than 100 modified (-M 100), and we eliminated oligotypes that appeared in less than three samples (-s 3). These filters removed 1.63% of *Prevotella* reads and 7.95 % of *Akkermansia* reads. We used LEfSe as described above to identify oligotypes (strains) associated with diet and/or geography (Table S1H). Based on the 11 high-entropy positions in the *Akkermansia* alignment, we constructed a Neighbour-Joining tree of the 7 oligotypes (strains).

*Short-chain fatty-acid (SCFA) analysis*

Phosphoric acid was purchased from Fisher Scientific. Ethyl acetate anhydrous (99.8%) and all the 99% grade standards (acetic acid, propionic acid, isobutyric acid, butyric acid, isovaleric acid, valeric acid and internal standard 4-methyl valeric acid) were purchased from Sigma-Aldrich.

Short chain fatty acids were extracted according to a protocol previously published by García‑Villalba *et al.* (21). Right after collection, faeces were weighed and 1 mL of 0.5% phosphoric acid was added per 100 mg of material. The suspensions were frozen at -20°C until extraction. Once thawed, faecal suspensions were homogenized 2 min with a vortex mixer then centrifuged 10 min at 17949 g at 4°C. Supernatant was collected and an equal volume of ethyl acetate was added. To extract SCFAs, samples were once again homogenized 2 min with a vortex mixer and centrifuged 10 min at 17949 g at 4°C. The organic phase was transferred to an autosampler vial and 4-methylvaleric acid added to obtain 500 µM of internal standard.

SCFAs were analysed with a GC-FID system (Shimadzu), constituted of a GC‑2010 Plus gas chromatograph equipped with an AOC-20s auto-sampler, an AOC-20i auto-injector and a flame ionisation detector. The system was controlled by the GC solution software. A 1 µL sample was injected to the Nukol capillary GC column (30 m x 0.25 mm id, 0.25 µM film thickness, Supelco analytical) with a split ratio of 1:10 (v/v). The column flow was constant at 1.2 mL/min of hydrogen. Nitrogen (30 mL/min), hydrogen (40 mL/min) and air (400 mL/min) were used as auxiliary gases for flame ionisation detector. The injector was set on 230°C and the detector on 250°C. The oven temperature was initially programmed at 100°C, then increased to 200°C at 8°C/min and maintained for 2 min at this temperature (total run time 14.5 min and 3 min equilibration).

*Data availability*

Raw 16S rRNA gene sequences have been deposited in the Qiita (<http://qiita.microbio.me/)> under Study ID 10439 and are available on GitHub (<https://github.com/cgir/16S_inuitgut>).

Supplementary References

1. **Statistics Canada**. 2011. 2011 National Household Survey.

2. **David LA**, **Maurice CF**, **Carmody RN**, **Gootenberg DB**, **Button JE**, **Wolfe BE**, **Ling A V**, **Devlin a S**, **Varma Y**, **Fischbach M a**, **Biddinger SB**, **Dutton RJ**, **Turnbaugh PJ**. 2014. Diet rapidly and reproducibly alters the human gut microbiome. Nature **505**:559–63.

3. **Preheim SP**, **Perrott AR**, **Martin-Platero AM**, **Gupta A**, **Alm EJ**. 2013. Distribution-based clustering: Using ecology to refine the operational taxonomic unit. Appl Environ Microbiol **79**:6593–6603.

4. **Caporaso JG**, **Lauber CL**, **Walters WA**, **Berg-Lyons D**, **Lozupone CA**, **Turnbaugh PJ**, **Fierer N**, **Knight R**. 2011. Global patterns of 16S rRNA diversity at a depth of millions of sequences per sample. Proc Natl Acad Sci USA **108**:4516–4522.

5. **Caporaso JG**, **Kuczynski J**, **Stombaugh J**, **Bittinger K**, **Bushman FD**, **Costello EK**, **Fierer N**, **Peña AG**, **Goodrich JK**, **Gordon JI**, **Huttley GA**, **Kelley ST**, **Knights D**, **Koenig JE**, **Ley RE**, **Lozupone CA**, **Mcdonald D**, **Muegge BD**, **Pirrung M**, **Reeder J**, **Sevinsky JR**, **Turnbaugh PJ**, **Walters WA**, **Widmann J**, **Yatsunenko T**, **Zaneveld J**, **Knight R**. 2010. QIIME allows analysis of high- throughput community sequencing data. Nat Methods **7**:335–336.

6. **Rideout JR**, **He Y**, **Navas-molina JA**, **Walters WA**, **Ursell LK**, **Gibbons SM**, **Chase J**, **Mcdonald D**, **Gonzalez A**, **Robbins-pianka A**, **Clemente JC**. 2014. Subsampled open-reference clustering creates consistent, comprehensive OTU definitions and scales to billions of sequences. PeerJ **2**:e545.

7. **McMurdie PJ**, **Holmes S**. 2013. Phyloseq: An R Package for Reproducible Interactive Analysis and Graphics of Microbiome Census Data. PLoS One **8**:e61217.

8. **R Development Core Team**. 2011. R: A language and environment for statistical computing. R Found Stat Comput.

9. **Wickham H**. 2009. ggplot2: elegant graphics for data analysisSpringer. New York.

10. **Lozupone C**, **Knight R**. 2005. UniFrac : a New Phylogenetic Method for Comparing Microbial Communities UniFrac : a New Phylogenetic Method for Comparing Microbial Communities. Appl Environ Microbiol **71**:8228–8235.

11. **Oksanen J**, **Blanchet F.**, **Kindt R**, **Legendre P**, **Minchin PR**, **O’Hara RB**, **Simpson GL**, **Solymos P**, **Stevens MHH**, **Wagner H**. 2015. vegan: Community Ecology Package. R package version 2.2-1.

12. **Tibshirani R**, **Walther G**, **Hastie T**. 2001. Estimating the number of clusters in a data set via the gap statistic. J R Stat Soc Ser B (Statistical Methodol **63**:411–423.

13. **De Filippo C**, **Cavalieri D**, **Di Paola M**, **Ramazzotti M**, **Poullet JB**, **Massart S**, **Collini S**, **Pieraccini G**, **Lionetti P**. 2010. Impact of diet in shaping gut microbiota revealed by a comparative study in children from Europe and rural Africa. Proc Natl Acad Sci USA **107**:14691–6.

14. **Clemente JC**, **Pehrsson EC**, **Blaser MJ**, **Sandhu K**, **Gao Z**, **Wang B**, **Magris M**, **Hidalgo G**, **Contreras M**, **Noya-Alarcon O**, **Lander O**, **McDonald J**, **Cox M**, **Walter J**, **Oh PL**, **Ruiz JF**, **Rodriguez S**, **Shen N**, **Song SJ**, **Metcalf J**, **Knight R**, **Dantas G**, **Dominguez-Bello MG**. 2015. The microbiome of uncontacted Amerindians. Sci Adv **1**:e1500183–e1500183.

15. **Schnorr SL**, **Candela M**, **Rampelli S**, **Centanni M**, **Consolandi C**, **Basaglia G**, **Turroni S**, **Biagi E**, **Peano C**, **Severgnini M**, **Fiori J**, **Gotti R**, **De Bellis G**, **Luiselli D**, **Brigidi P**, **Mabulla A**, **Marlowe F**, **Henry AG**, **Crittenden AN**. 2014. Gut microbiome of the Hadza hunter-gatherers. Nat Commun **5**:3654.

16. **Segata N**, **Izard J**, **Waldron L**, **Gevers D**, **Miropolsky L**, **Garrett WS**, **Huttenhower C**. 2011. Metagenomic biomarker discovery and explanation. Genome Biol **12**:1.

17. **Love MI**, **Huber W**, **Anders S**. 2014. Moderated estimation of fold change and dispersion for RNA-Seq data with DESeq2. Genome Biol **15**:1–21.

18. **Eren AM**, **Morrison HG**, **Lescault PJ**, **Reveillaud J**, **Vineis JH**, **Sogin ML**. 2015. Minimum entropy decomposition: unsupervised oligotyping for sensitive partitioning of high-throughput marker gene sequences. ISME J **9**:968–979.

19. **Eren AM**, **Sogin ML**, **Morrison HG**, **Vineis JH**, **Fisher JC**, **Newton RJ**, **McLellan SL**. 2015. A single genus in the gut microbiome reflects host preference and specificity. ISME J **9**:90–100.

20. **Eren AM**, **Maignien L**, **Sul WJ**, **Murphy LG**, **Grim SL**, **Morrison HG**, **Sogin ML**. 2013. Oligotyping: Differentiating between closely related microbial taxa using 16S rRNA gene data. Methods Ecol Evol **4**:1111–1119.

21. **García-Villalba R**, **Giménez-Bastida JA**, **García-Conesa MT**, **Tomás-Barberán FA**, **Carlos Espín J**, **Larrosa M**. 2012. Alternative method for gas chromatography-mass spectrometry analysis of short-chain fatty acids in faecal samples. J Sep Sci **35**:1906–1913.
